# Influence of Coronary Flow and Left Ventricular Outflow Tract Velocity on LDL Accumulation and Calcification in Aortic Valve Leaflets

**DOI:** 10.3390/biomechanics5040099

**Published:** 2025-12-02

**Authors:** Mishal Raza-Taimuri, Ian Y. Chen, Hamid Sadat

**Affiliations:** 1 Department of Mechanical Engineering, University of North Texas, Denton, TX 76207, USA; 2 Department of Medicine, Division of Cardiovascular Medicine, Stanford Cardiovascular Institute, Stanford University, Palo Alto, CA 94304, USA

**Keywords:** CFD, fluid–structure interaction, aortic valve calcification, hemodynamics

## Abstract

**Background/Objectives:**

Calcific aortic valve disease (CAVD) is a progressive condition marked by thickening and calcification of the valve leaflets, leading to impaired cardiac function and increased cardiovascular risk. As disease progression is strongly influenced by hemodynamics and lipid accumulation, computational modeling provides a powerful tool for understanding the biomechanical drivers of calcification.

**Methods:**

This study investigates the effects of coronary artery flow and varying left ventricular outflow tract (LVOT) velocity profiles on low density lipoprotein (LDL) accumulation and associated aortic valve calcification using a partitioned fluid–structure interaction framework coupled with scalar transport modeling, with a focus on understanding the differential behaviors of the three valve leaflets: the non-coronary cusp (NCC), right coronary cusp (RCC), and left coronary cusp (LCC). Four distinct LVOT flow velocity profiles (anterior, lateral, posterior, and medial) and coronary flow are simulated to determine their effects on the distribution of LDL accumulation and associated calcification across the valve leaflets.

**Results/Conclusions:**

Our results indicate that the RCC experiences greatest excursion and lowest calcification. The LCC shows lowest excursion and slightly higher susceptibility for calcification. Finally, the NCC experiences intermediate excursion, but is most prone to calcification.

## 1. Introduction

Calcific aortic valve disease (CAVD) is the most common valvular disorder worldwide, characterized by progressive thickening, fibrosis, and calcification of the aortic valve leaflets, which impair cardiac function [1]. The disease begins with endothelial damage and lipid infiltration, triggering chronic inflammation and the activation of valvular interstitial cells (VICs). These cells transform into osteoblast-like cells that deposit calcium, forming nodules that stiffen the valve leaflets and obstruct blood flow. This progression leads to aortic stenosis, a condition associated with severe cardiovascular complications such as heart failure, which has significantly increased morbidity and mortality [2]. In fact, CAVD-related mortality has nearly doubled worldwide in the past 30 years, reaching approximately 127,000 deaths [3]. Given the complex relationship between hemodynamics, leaflet mechanics, and disease progression, advanced computational techniques are essential for studying the impact of calcification on valve function and guiding the development of treatment strategies.

Computational modeling, specifically with the use of fluid structure interaction (FSI), has emerged as a powerful tool to study the biomechanical and hemodynamic impacts of calcification on aortic valve function. Among these models, the use of uniform prescribed calcification approaches is prevalent where calcified regions are predefined based on idealized patterns, allowing researchers to evaluate their impact on valve dynamics without simulating biological drivers like lipid transport or inflammation [4–10].

Although these studies have provided valuable insights into the impact of prescribed calcification on valve function, they typically overlook the heterogeneity observed in reality. Most assume uniform calcification distributions, without accounting for the spatially non-uniform patterns often seen in patients.

Our group previously addressed this limitation by simulating cases with varying calcification along the leaflet length, and we found that such distributions significantly increased vortex shedding and elevated WSS on the ventricular side of the leaflet [11]. We also found that non-uniformly calcified cases were misclassified by most clinical diagnostic techniques. Amindari et al. [12] conducted a similar analysis by non-linearly changing hyperelastic material properties along the leaflet thickness and found that such anisotropic behavior resulted in stabilization of leaflet movements, thus reducing flutter. Xie et al. [13] studied a 3D valve with different levels of calcium deposition coverage, which were found to increase the tensional burden on leaflets.

However, these studies still employ prescribed calcification which restricts the ability to evaluate critical factors influencing CAVD, such as the impact of coronary artery flow. Chen et al. [14] clinically reported that the non-coronary cusp (NCC) tends to exhibit greater calcification compared to the left and right coronary cusps (LCC and RCC). Additionally, variations in left ventricular outflow tract (LVOT) velocity across the aortic cross-section may amplify these differences [15], hence adding further complexity to CAVD modeling [16–18].

To accurately predict calcification patterns, the calcification process itself must be considered, which is highly complex and influenced by multiple factors such as hemodynamic forces, leaflet dynamics and bio transport of various lipid components including lipoprotein(a), low-density lipoprotein (LDL), and triglyceride-rich lipoproteins [19]. Among these, LDL is widely regarded as one of the most important predictors of calcification progression. Several clinical studies have linked elevated LDL cholesterol penetration into tissue to the development and progression of CAVD. Demer and Tintut [19] demonstrated that once LDL penetrates into aortic tissue and becomes oxidized, it triggers inflammatory cascades and stimulates the transformation of valvular interstitial cells into osteoblast-like cells, thereby setting the stage for calcific remodeling of the valve. This highlights that once circulating LDL levels are elevated, they are more susceptible to penetration and oxidative modification, leading to increased lipid accumulation, calcific deposition, and consequent stiffening of the aortic valve [20]. Additionally, animal models of hypercholesterolemia (i.e., elevated levels of cholesterol in blood) have shown that elevated LDL can directly cause valve thickening and calcification [21].

In this study, a 2D aortic valve model was employed to explore how coronary artery dynamics and variations in left ventricular outflow tract (LVOT) velocity profiles, each representing distinct regions of the LVOT cross-section, influence leaflet biomechanics and calcification patterns in the left coronary cusp (LCC), right coronary cusp (RCC), and non-coronary cusp (NCC). Calcification was modeled by mapping local LDL accumulation onto leaflet elasticity.

## 2. Methodology

### 2.1. CFD Methodology

A partitioned FSI framework was employed to analyze the behavior of the aortic valve leaflets. The fluid side was solved using OpenFOAM [22], a finite volume-based solver, while the structural response was computed with CalculiX [23], a finite element-based solver. The coupling between the two solvers was facilitated by the multiphysics library, PreCICE [24].

#### 2.1.1. Fluid Flow Solver

To closely mimic physiological flow, the fluid domain was solved using OpenFOAM [22], which solves the incompressible Navier–Stokes equations for laminar flow [25,26] using the finite volume method. The equations are expressed in Arbitrary Lagrangian Eulerian (ALE) form to account for mesh deformation due to leaflet motion:

(1) ∇·(U)=0

(2) $$\frac{\partial U}{\partial t}+\left(U-U_{g}\right)\cdot \nabla U=-\frac{1}{\rho }\nabla P+v\Delta U$$

Equation (1) represents the continuity equation and (2) is the momentum equation where **U** is the fluid velocity, *t* is time, $U_{g}$ is the grid point velocity, *ρ* is the fluid density, *P* is the pressure, and *ν* is the kinematic viscosity. Blood flow through the heart was modeled with a density of $1060\;\frac{kg}{m^{3}}$ and a dynamic viscosity of $0.0035\;\frac{kg}{\mathrm{m s}}$.

In order to model LDL transport, the following equation was also solved:

(3) $$\frac{\partial C}{\partial t}+\nabla \cdot \left(\left(U-U_{g}\right)C\right)=\nabla \cdot (D\nabla C)$$

where *C* is the scalar quantity being transported and *D* is the diffusion coefficient of the scalar in the fluid.

The PIMPLE algorithm, combining PISO and SIMPLE, is employed for pressure velocity coupling. To model dynamic mesh motion, a Radial Basis Function (RBF) mesh motion solver is employed which smoothly propagates boundary-node displacements to interior nodes via radial interpolation, preserving mesh quality during large leaflet movements and preventing mesh entanglement [27]. This study employs first-order explicit Euler for time discretization, a bounded upwind scheme for convection, and linear interpolation for diffusion. Gradient and Laplacian terms were discretized using a Gauss linear corrected scheme, with corrected surface-normal gradients.

#### 2.1.2. Structural Analysis Solver

CalculiX [23] was employed to solve the elastic deformation of the aortic valve leaflet using the finite element method in a Lagrangian framework. The governing equation of motion is:

(4) $$\rho \frac{\partial^{2}d}{\partial t^{2}}+\nabla \cdot \Sigma =0$$

where *d* is the displacement vector and Σ is the Cauchy stress tensor, defined for linear elastic materials as:

(5) $$\Sigma =2\mu^{\prime \prime }+\lambda tr{(}^{\prime \prime })I$$

(6) $$\prime \prime =\frac{1}{2}\left(\nabla d+\nabla d^{⊤}\right)$$

Here, **I** is the second-rank identity tensor and (μ,λ) are the Lamé parameters, computed from the Young’s modulus *E* and Poisson’s ratio *ν* as $\mu =\frac{E}{2(1+v)}$ and $\lambda =\frac{vE}{\left(1+v)(1-2v\right)}$.

The leaflets were modeled as isotropic linear elastic structures, which is a commonly used approach because it provides a computationally efficient approximation of valve mechanics while capturing key deformation characteristics [28,29]. A density of $1060\;\frac{kg}{m^{3}}$ and Poisson’s ratio of 0.3 are assigned [6,9]. The initial Young’s modulus was set to 2 MPa, representative of healthy tissue [9], and was spatially updated in calcified regions based on predicted LDL accumulation, as described in Section 2.3. A dynamic explicit formulation was used.

#### 2.1.3. Fluid–Structure Interaction

A partitioned two-way FSI approach was adopted using the multiphysics coupling library preCICE [24]. The fluid and solid solvers were coupled using a parallel implicit scheme accelerated by the Interface Quasi-Newton Inverse Least-Squares (IQN-ILS) method with an initial relaxation factor of 0.1. The coupling interface exchanged fluid forces and structural displacements between the domains. Nearest-neighbor mapping was used to transfer values between non-matching grids by associating each node with its closest counterpart, with a consistent mapping constraint applied in both directions.

Coupling convergence was enforced using a relative residual tolerance of 5 × 10^−3^ [30–32], for both displacement and force, with a maximum of 50 coupling iterations per time step. A uniform FSI time step of 0.0001 s was used throughout the simulation.

### 2.2. Geometry and Grid

**Figure 1.**
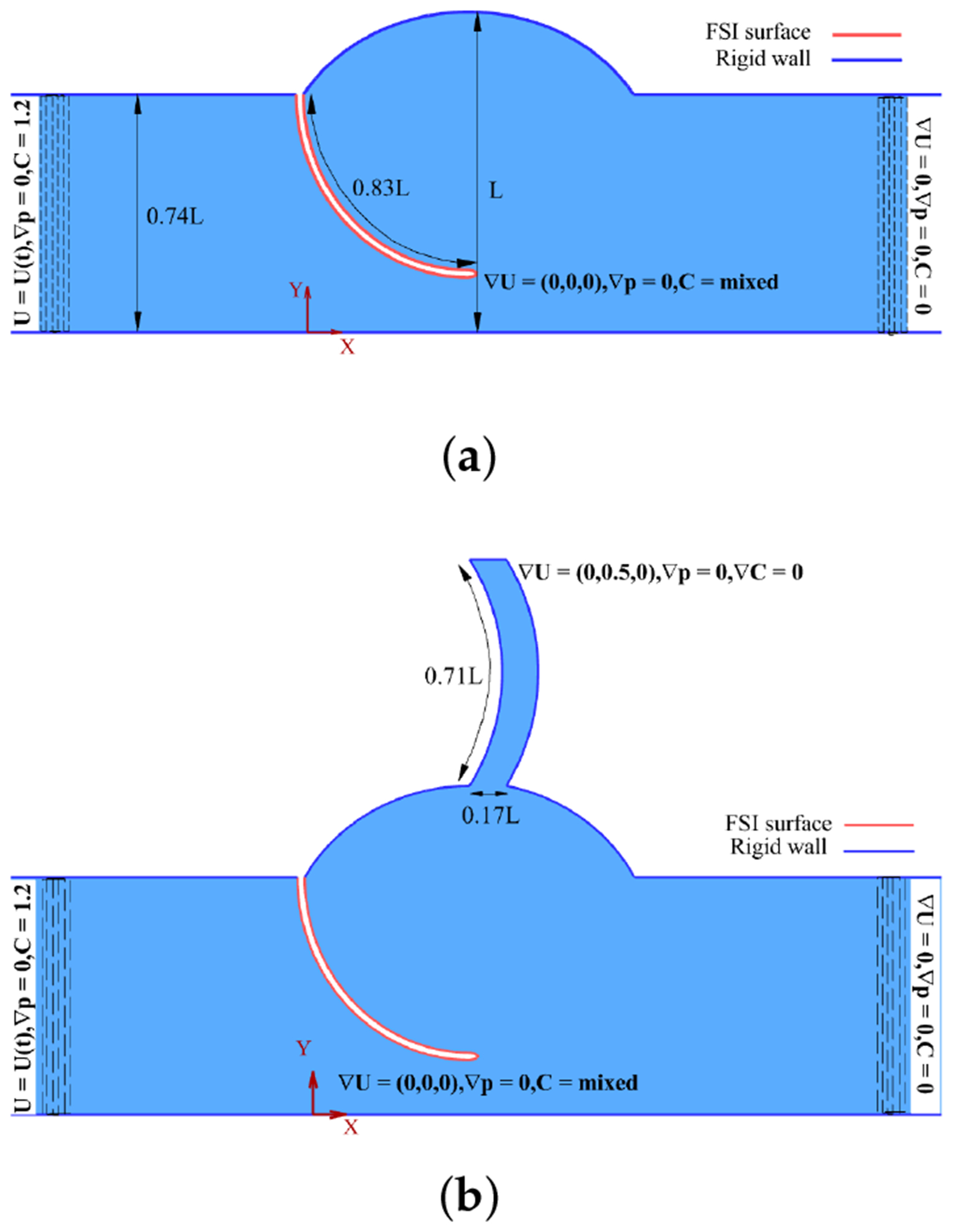
Configurations of the symmetric 2D aortic valve geometry. (**a**) Without coronary artery. (**b**) With coronary artery.

A dimensionless 2D aortic valve model was derived from CT images [14] and literature measurements, then constructed in GMSH [33] with two cusps and a housing. To enhance computational efficiency, a half-domain with symmetry along the mid-plane was used (Figure 1a). Leaflets were assigned a thickness of 0.02 L, where *L* = 0.0175 m, with the origin positioned below the cusp attachment and the domain extended 2.3 L upstream and 4.1 L downstream of the valve. A modified geometry incorporating a coronary outlet was used to assess its influence on flow (Figure 1b). In both models, the leaflet acts as the FSI interface (red surface), while the remaining boundaries are treated as rigid walls, as several studies have shown that wall elasticity has a negligible impact on valve behavior [4,6,29,34].

The fluid domain uses a 3D tetrahedral mesh which includes 40 cells along the leaflet and tract walls, 80 along the sinus and tract boundaries, 300 across the sinus width, all set to be one-cell thick in the Z-direction (depth), to mimic a 2D simulation. This mesh ensures more robust handling of large leaflet motion [35]. The grid was carefully designed to resolve steep gradients in both velocity and scalar concentration near the walls and leaflet surfaces, ensuring adequate boundary layer resolution.

**Figure 2.**
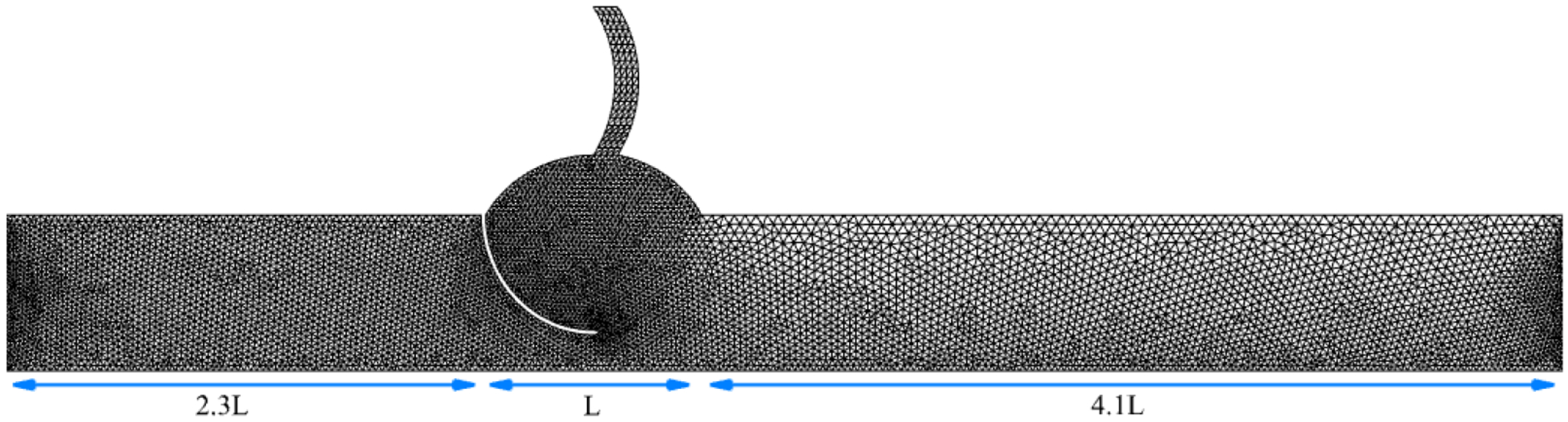
Fluid mesh domain and grid with coronary artery.

For grid verification, two additional meshes with a 1.25 refinement factor were generated. The final fluid mesh for the coronary case is shown in Figure 2. The solid leaflet is modeled with a one-cell-thick mesh in the z-direction, using $\tilde{4}0$ C3D8 hexahedral elements.

### 2.3. Boundary Conditions

**Figure 3.**
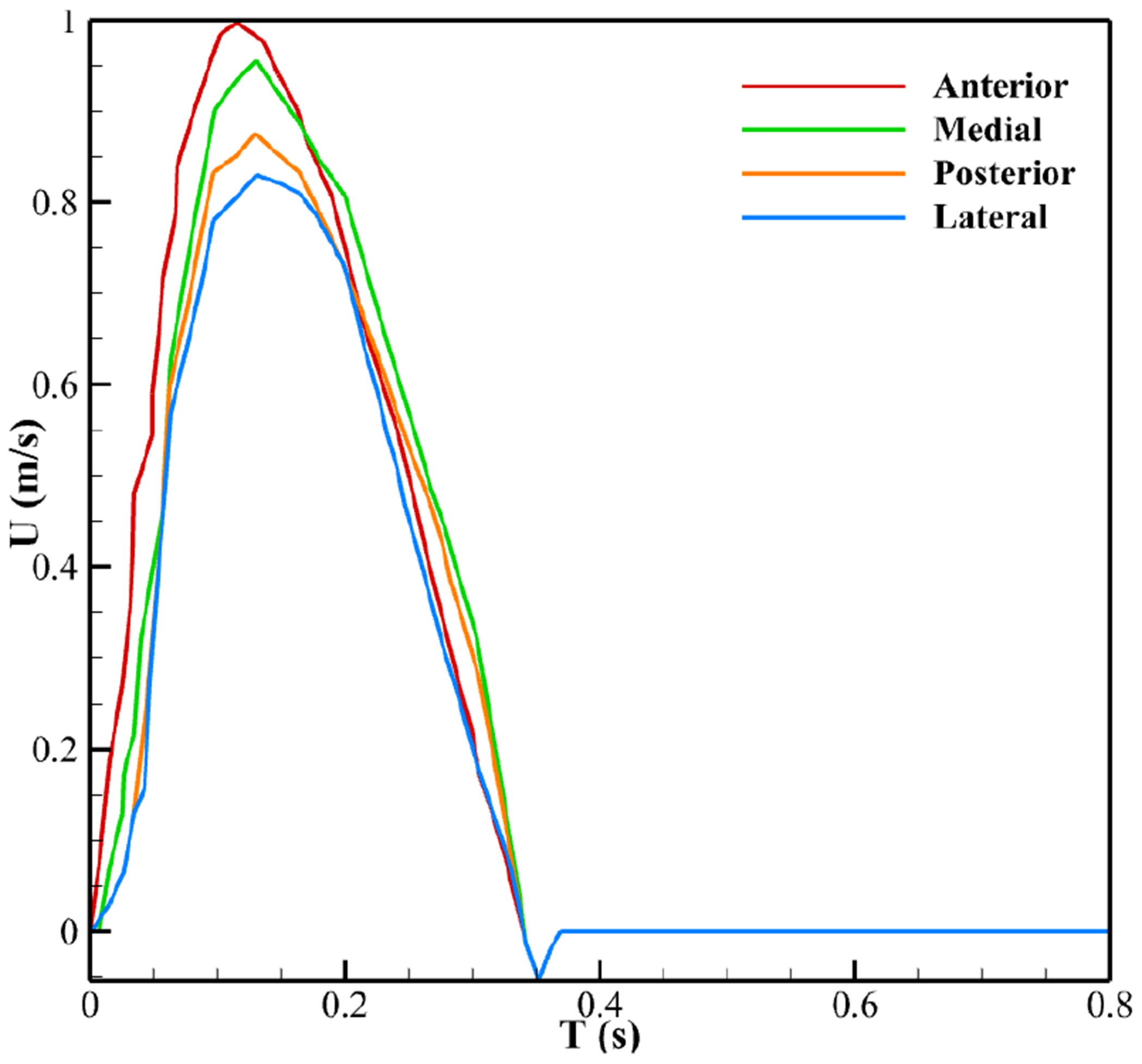
Pulsatile inlet velocity profiles.

**Figure 4.**
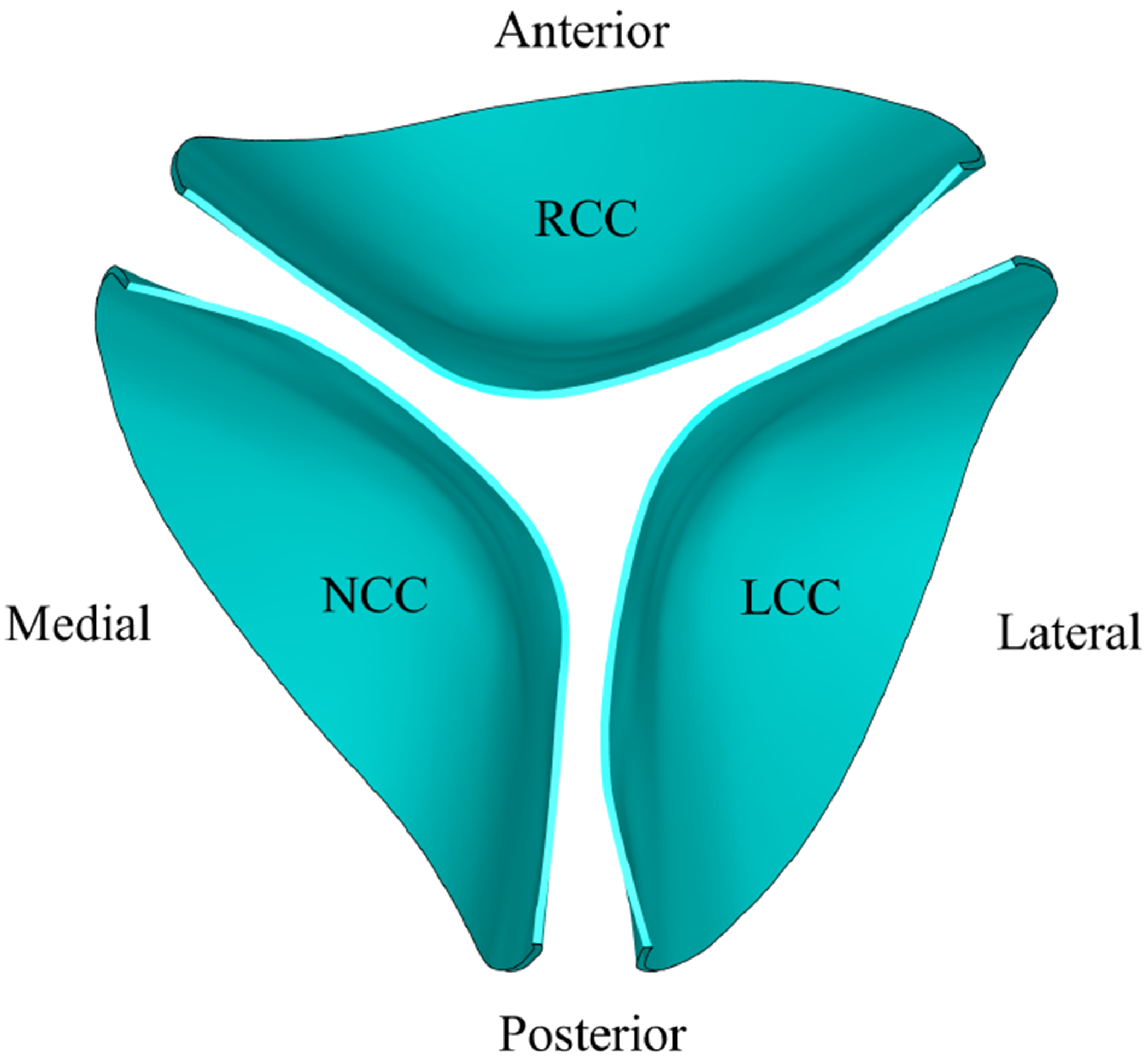
Location of the three leaflets in the aortic orifice.

To study the effect of velocity on LDL accumulation patterns, transient, unsteady FSI simulations were performed over the 0.8-s cardiac cycle, with four time-dependent LVOT velocity profiles (Figure 3) applied at the aortic valve inlet, as reported by Amindari et al. [6] and Kupari et al. [15]. Each profile represents a distinct region of the aortic cross-section (anterior, lateral, medial, and posterior) and corresponds to the inflow for the RCC, LCC, and NCC, respectively, as presented by Kupari et al. [15] and illustrated in Figure 4.

For the case with a coronary artery, an outlet velocity boundary condition was applied at the coronary outlet with a fixed value of 0.5 m/s, as adopted in several computational studies [36,37], to represent the time-averaged coronary flow over the cardiac cycle [38]. At the aortic outlet, a pressure outlet boundary condition was imposed with a zero-gradient condition on velocity. A symmetry boundary condition was applied at the mid-plane while all other surfaces were assigned a no-slip condition.

To investigate the LDL accumulation on the leaflet, a mixed boundary condition was implemented on the leaflet surface following the equation for large and medium sized arteries [39–41]

(7) $$V_{w}C_{w}-{\left.D\frac{\partial C}{\partial n}\right|}_{\text{wall}}\;=KC_{w}\;\text{at the wall}$$

where $V_{w}$ is the plasma filtration velocity normal to the wall set to 4 × 10^−8^ m/s, *C_w_* is the endothelial surface concentration of LDL, *n* is the unit vector normal to the wall and *K* is the physiological endothelial permeability of LDL assigned as 2 × 10^−10^ m/s [39,42]. *D* is the physiological LDL diffusivity, usually set to 5 × 10^−12^ m^2^/s [40]. However, this value yields a Schmidt number of approximately 5 × 10^5^, resulting in a very thin concentration boundary layer that requires fine mesh resolution, which increases computational cost and can potentially cause mesh entanglement. Additionally, it leads to a high Peclet number, which can introduce numerical instability. To address these challenges, a diffusivity of 1 × 10^−10^ m^2^/s was employed to thicken the scalar boundary layer and facilitate faster, more stable computations.

An LDL concentration of *C* = 1.2 mg/mL was set at the inlet while all other boundaries were set to a zero gradient boundary condition [43–46]. LDL accumulation patterns on leaflet were integrated into the FSI framework to alter leaflet elasticity and assess their role in driving calcification. Non-calcified regions with a C of 1.2 mg/mL [39] were adjusted to have an E of 2 MPa, while severely calcified regions, with a C of 1.6 mg/mL as indicated in the literature [47] and supported by others [4,6,34], were adjusted to an E of 20 MPa. E values for any predicted C values between these two were modulated based on a linear scaling.

## 3. Results

### 3.1. Verification

**Figure 5.**
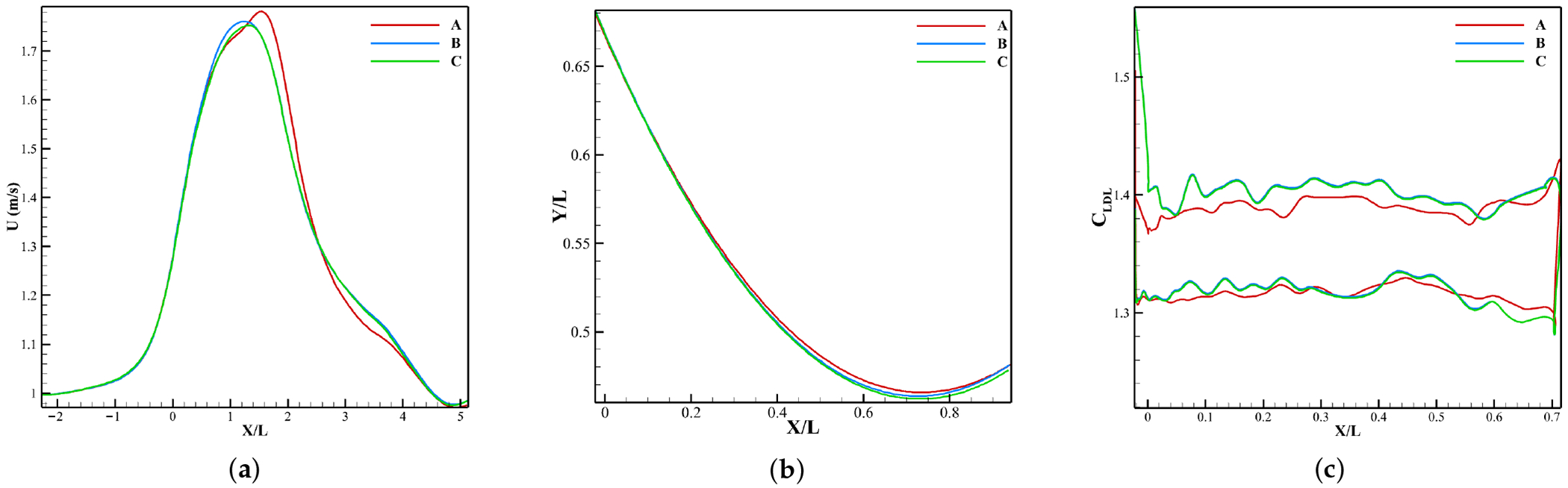
Grid verification at peak systole. (**a**) centerline stream-wise velocity from inlet to outlet. (**b**) Leaflet profile. (**c**) LDL accumulation variation along leaflet surface.

An FSI mesh convergence study was conducted using coarse (A), medium (B) and fine (C) meshes for the normal case with no coronary artery. The *U_x_* profile along a center line from inlet to outlet, the leaflet profile and the LDL concentration on the leaflet surface were all plotted at peak systole as shown in Figure 5.

Mesh A results in different velocities (Figure 5a) beyond the leaflet attachment (*X*/*L* > 0) compared to B and C, which show almost identical variation in streamwise velocity. This altered behavior in the flow field for mesh A changes leaflet dynamics especially near the belly and tip regions, as seen in Figure 5b, compared to the two finer meshes. As a result of the above two, the LDL accumulation (Figure 5c) has overall different values for mesh A but becomes more consistent and similar for meshes B and C. In light of these observations and to ensure grid independence, Mesh C, which is the finest mesh, is used in this study.

### 3.2. Effect of Coronary Artery on LDL Distribution of Aortic Valve Leaflets

To investigate the effect of coronary artery (CA) presence on calcification patterns, simulations were first performed using the anterior inlet velocity profile (Figure 3) to characterize LDL distributions with and without a coronary artery. These LDL patterns were then used to model the corresponding calcification on the aortic valve surface.

#### 3.2.1. Velocity Field and LDL Distribution

**Figure 6.**
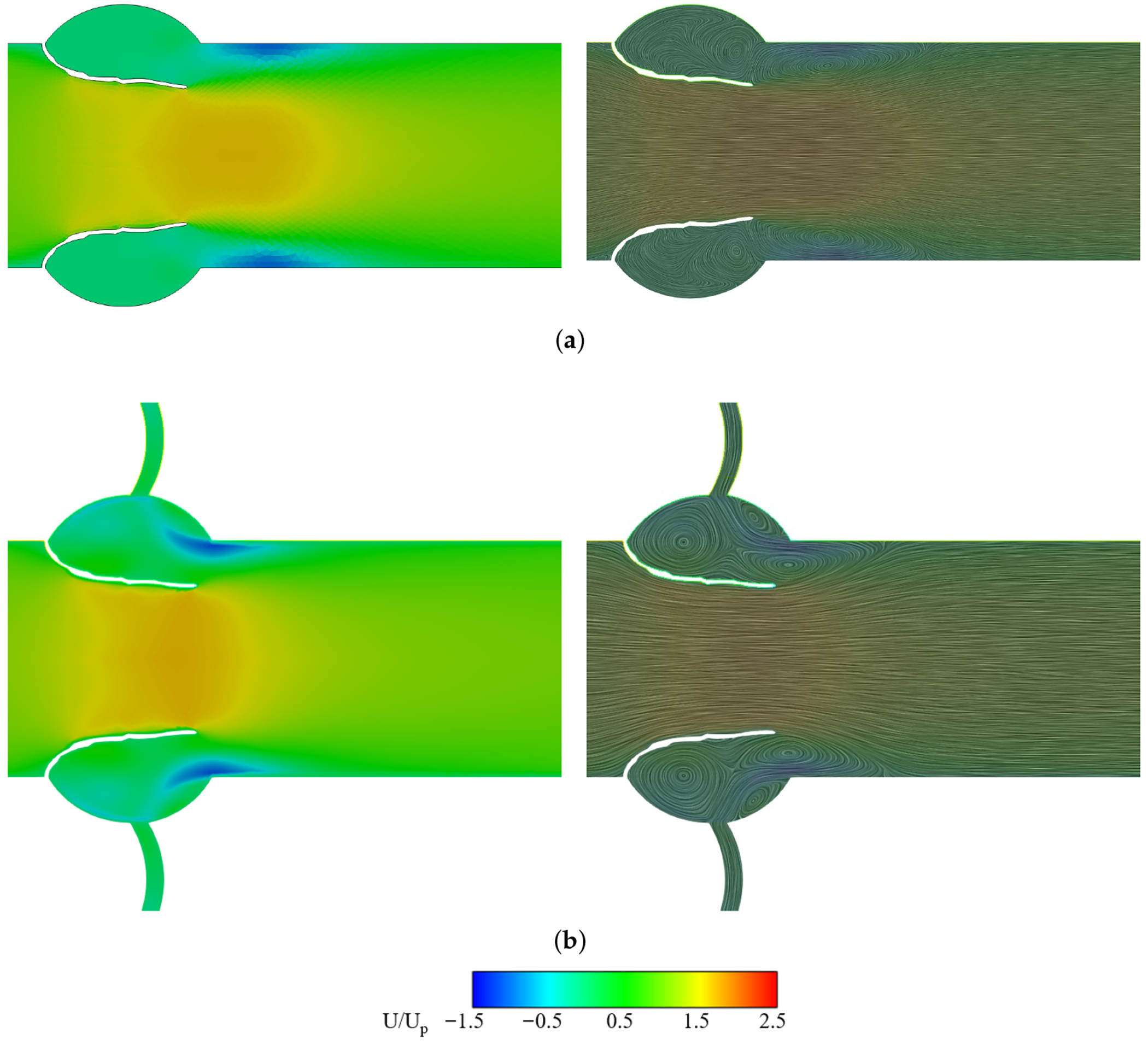
Velocity contours and streamlines at peak systole. (**a**) non-calcified without CA. (**b**) non-calcified with CA.

Figure 6 shows normalized streamwise velocity contours (*U*/*U_p_*) for non-calcified (normal) valves without and with a coronary artery, respectively, where *U_p_* is the peak velocity. Without CA, flow forms a distinct high-velocity jet across the aortic orifice, followed by downstream separation and circulation along the outflow tract walls. CA presence results in a shorter and wider velocity jet as flow diverts into the coronary outlet with enhanced circulation on the aortic side of the leaflets.

**Figure 7.**
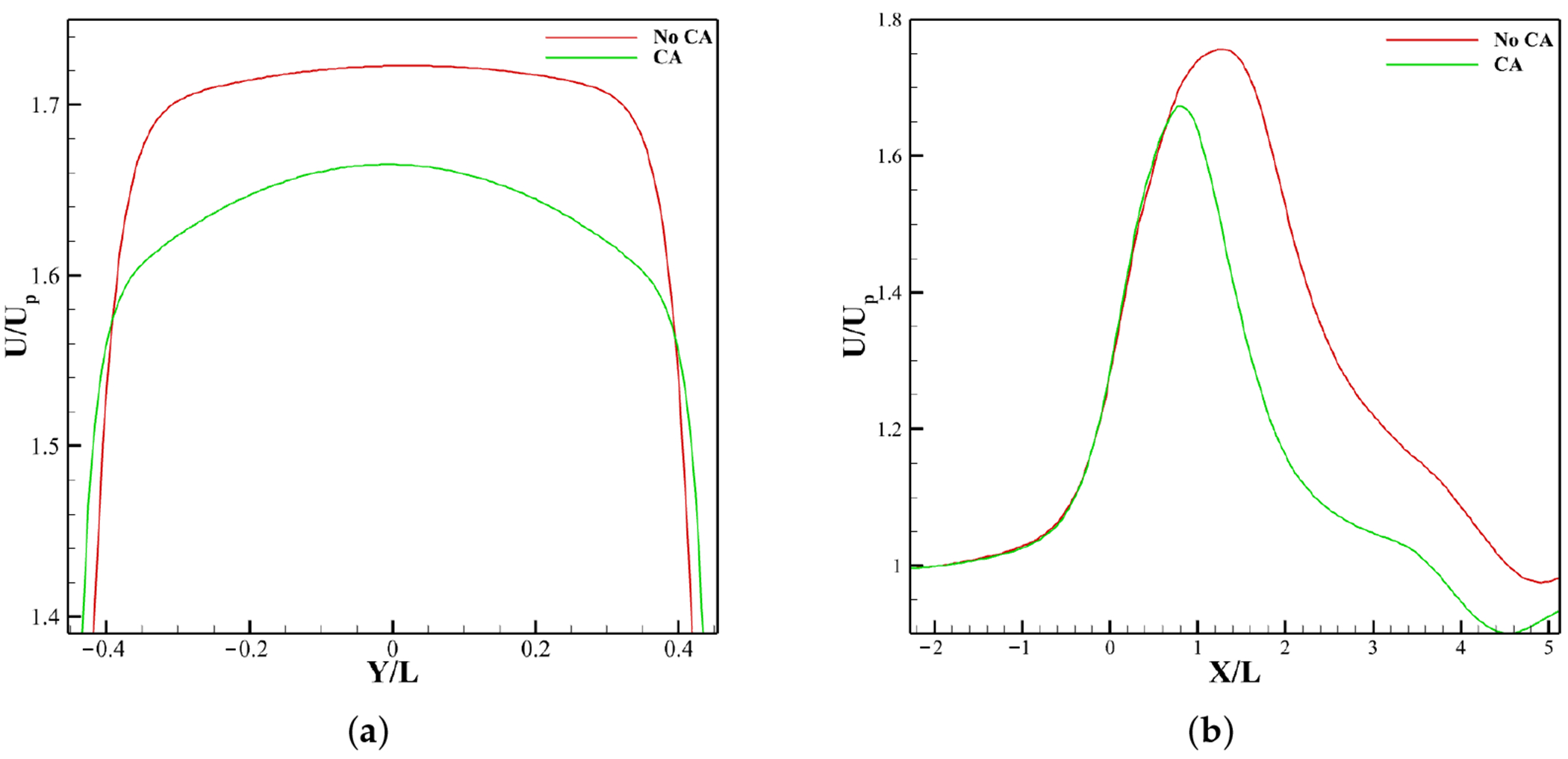
Velocity analysis for different non-calcified cases at peak systole. (**a**) Jet velocity at aortic orifice. (**b**) Streamwise velocity from inlet to outlet.

This trend is corroborated in Figure 7a, where the jet velocity between the leaflet tips is higher for the no CA case than for the CA case. The centerline velocity from inlet to outlet (Figure 7b) rises near the attachment point (*X*/*L* ≈ 0) in both cases, but peaks higher and later without CA, before gradual recovery downstream.

**Figure 8.**
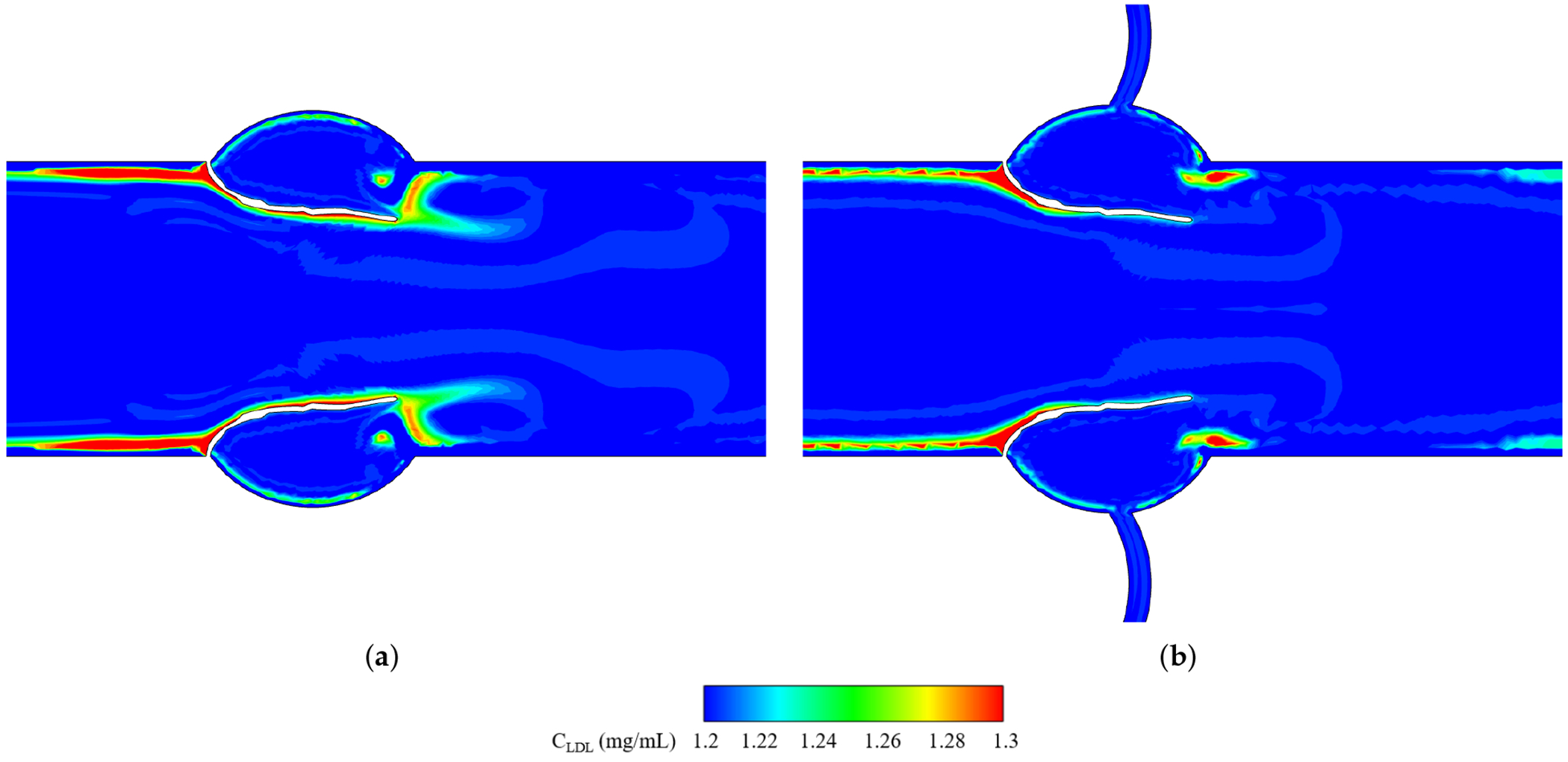
Contours of LDL accumulation in the leaflet vicinity at peak systole. (**a**) non-calcified without CA. (**b**) non-calcified with CA.

Figure 8 shows LDL accumulation contours at peak systole for non-calcified valves with and without CA. *C*_LDL_ in the field is about 1.2 mg/mL, but values change near the leaflet due to the imposed boundary condition (see Equation (7)). Globally, accumulation is greater on the ventricularis (ventricular side) than on the fibrosa (aortic side), consistent with Sadrabadi et al. [41]. Locally, the ventricular side shows non-uniform patterns, peaking near the attachment and decreasing toward the tip.

**Figure 9.**
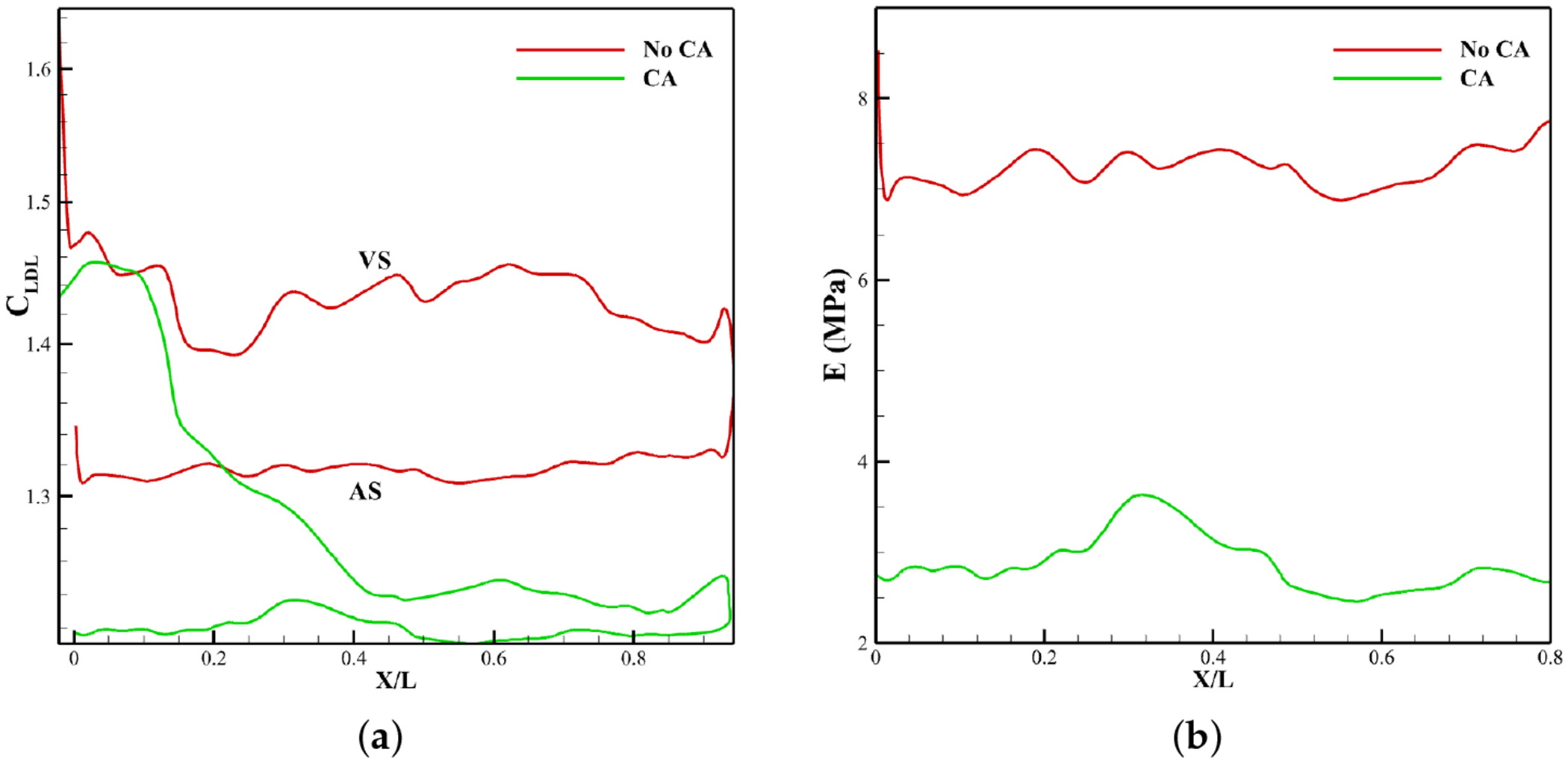
LDL accumulation and calcification along the leaflet’s ventricular side (VS) and aortic side (AS). (**a**) LDL accumulation pattern along leaflet. (**b**) Elastic Modulus along calcified leaflet.

Without CA, *C*_LDL_ peaks near the attachment (*X*/*L* ≈ 0) on the ventricular side (Figure 9a) and decreases toward the tip, while the aortic side remains mildly elevated but uniform. With CA, enhanced circulation lowers LDL levels on both sides (Figure 6); the ventricular side approaches baseline toward the tip, and the aortic side stays nearly uniform along its surface.

LDL distributions on the aortic side were converted into changes in leaflet elasticity to define calcification patterns (Section 2.3), since this side is more susceptible to calcification which is not only supported by Weinberg et al. [48], Yip et al. [49], and Sadrabadi et al. [41] but is also confirmed by the WSS on the aortic side, which is one order lower compared to the ventricular side (Figure 15b), as discussed later. The resulting Young’s modulus distributions from attachment to tip are shown in Figure 9b.

**Figure 10.**
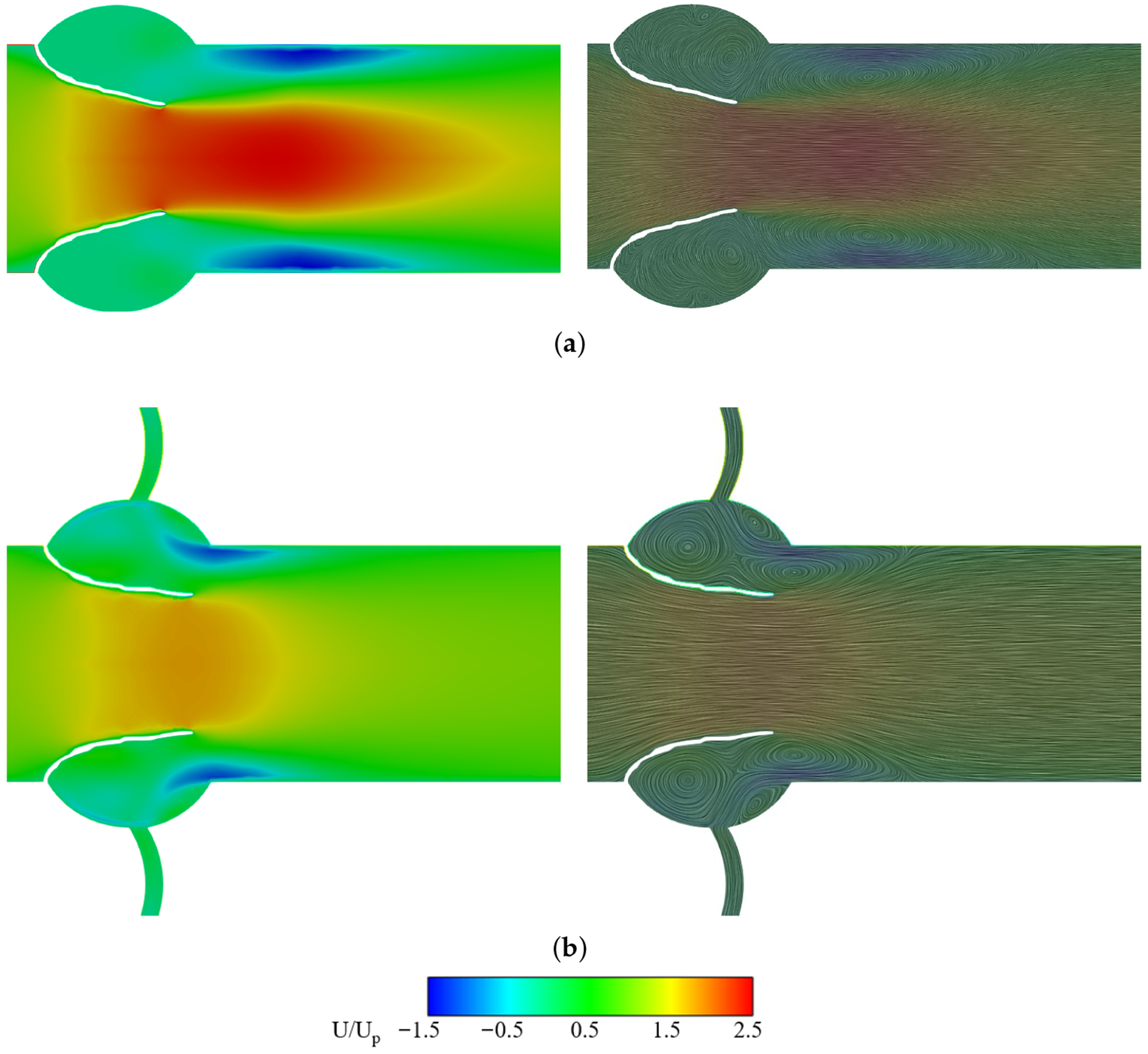
Velocity contours and streamlines at peak systole. (**a**) Calcified without CA. (**b**) Calcified with CA.

**Figure 11.**
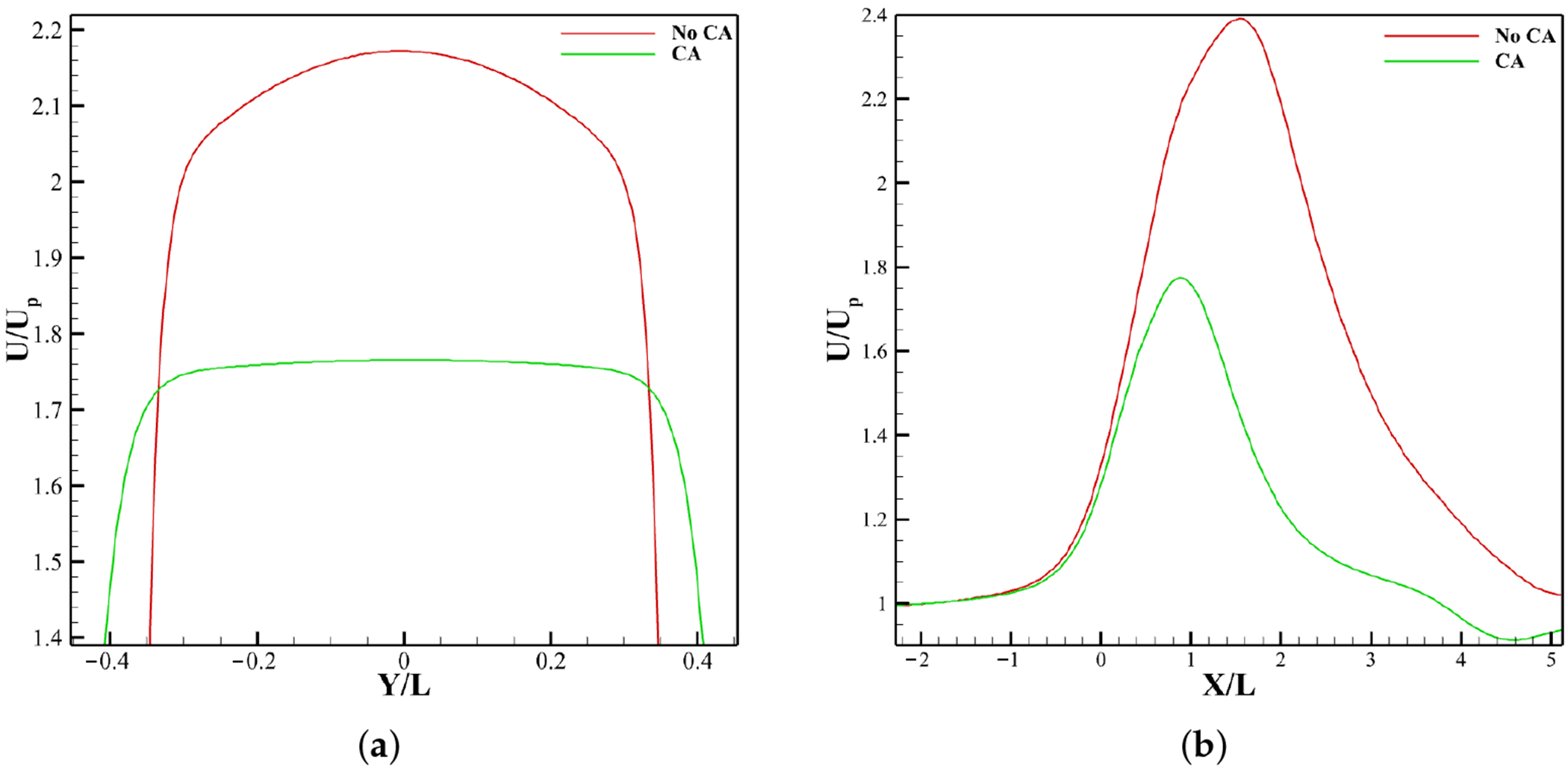
Velocity analysis for different calcified cases at peak systole. (**a**) Jet velocity at aortic orifice; (**b**) Streamwise velocity from inlet to outlet.

The velocity contours for calcified leaflets reveal flow alterations due to LDL-induced stiffening (Figure 10). In the calcified no CA case, orifice constriction produces a high-velocity jet (Figure 11a) stronger than its corresponding non-calcified case, extending farther downstream with a higher streamwise velocity spike and delayed recovery (Figure 11b), accompanied by wider regions of flow separation and circulation. In contrast, the calcified CA case shows minor constriction and a flow pattern similar to the non-calcified CA condition, with a slightly stronger jet but comparable recovery and limited separation.

#### 3.2.2. Vorticity and Pressure

**Figure 12.**
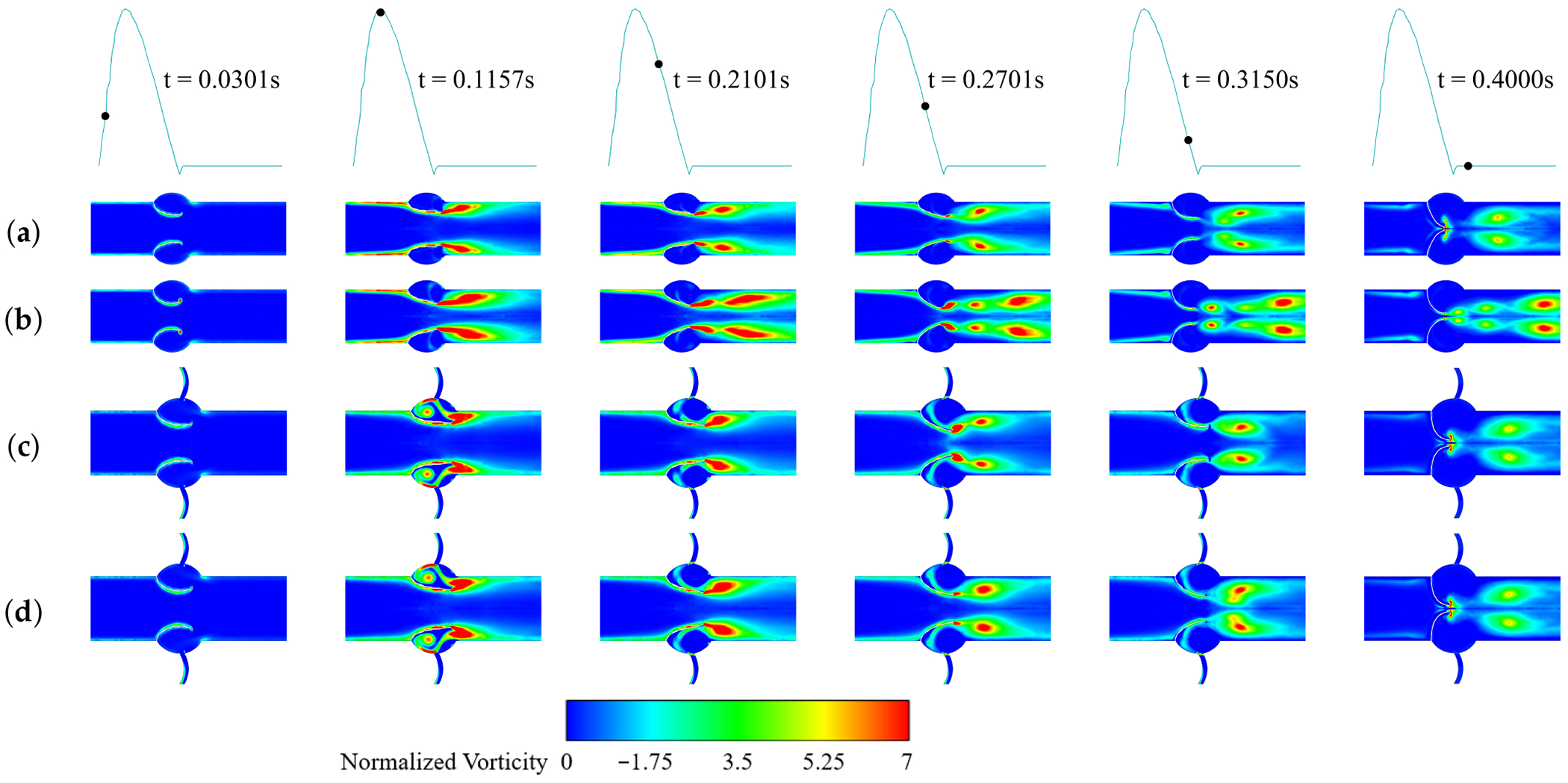
Normalized vorticity for different cases: (**a**) Non-calcified without CA; (**b**) Calcified without CA; (**c**) Non-calcified with CA; (**d**) Calcified with CA.

Figure 12 illustrates the temporal evolution of normalized vorticity contours throughout the cardiac cycle. For the normal no-CA case, cusp opening initiates vortex formation at the aortic orifice and shear layers along the boundaries. At peak systole, vortices detach and dissipate downstream, and after systole a wake of alternating vortices develops, indicating increased mixing and dissipation. Toward diastole, leaflet closure suppresses flow and vortex shedding. Once calcified, vortices become larger and more intense, interacting strongly with the constricted jet and producing a chaotic wake with complex detachments and reattachments. With a CA, increased circulation is evident near the coronary outlet at peak systole, but vortex shedding at the leaflets is markedly reduced, with negligible shear layers. Even after calcification, the pattern remains similar to the normal CA case, with only a slight increase in shedding, indicating that coronary artery presence reduces flow disturbances in the leaflet vicinity.

**Figure 13.**
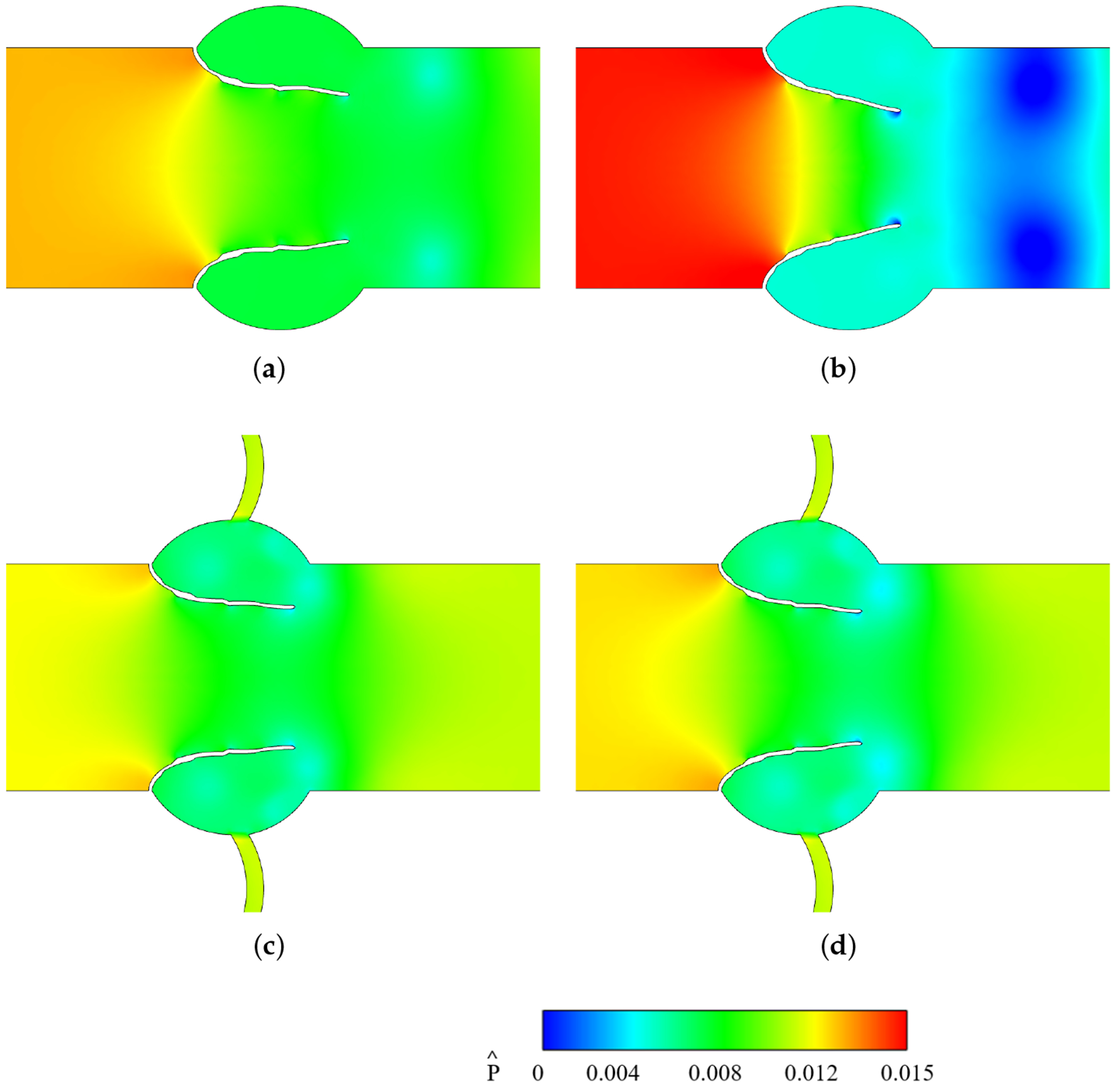
Non-dimensionalized pressure contours in the leaflet vicinity at peak systole. (**a**) Non-calcified without CA. (**b**) Calcified without CA. (**c**) Non-calcified with CA. (**d**) Calcified with CA.

**Figure 14.**
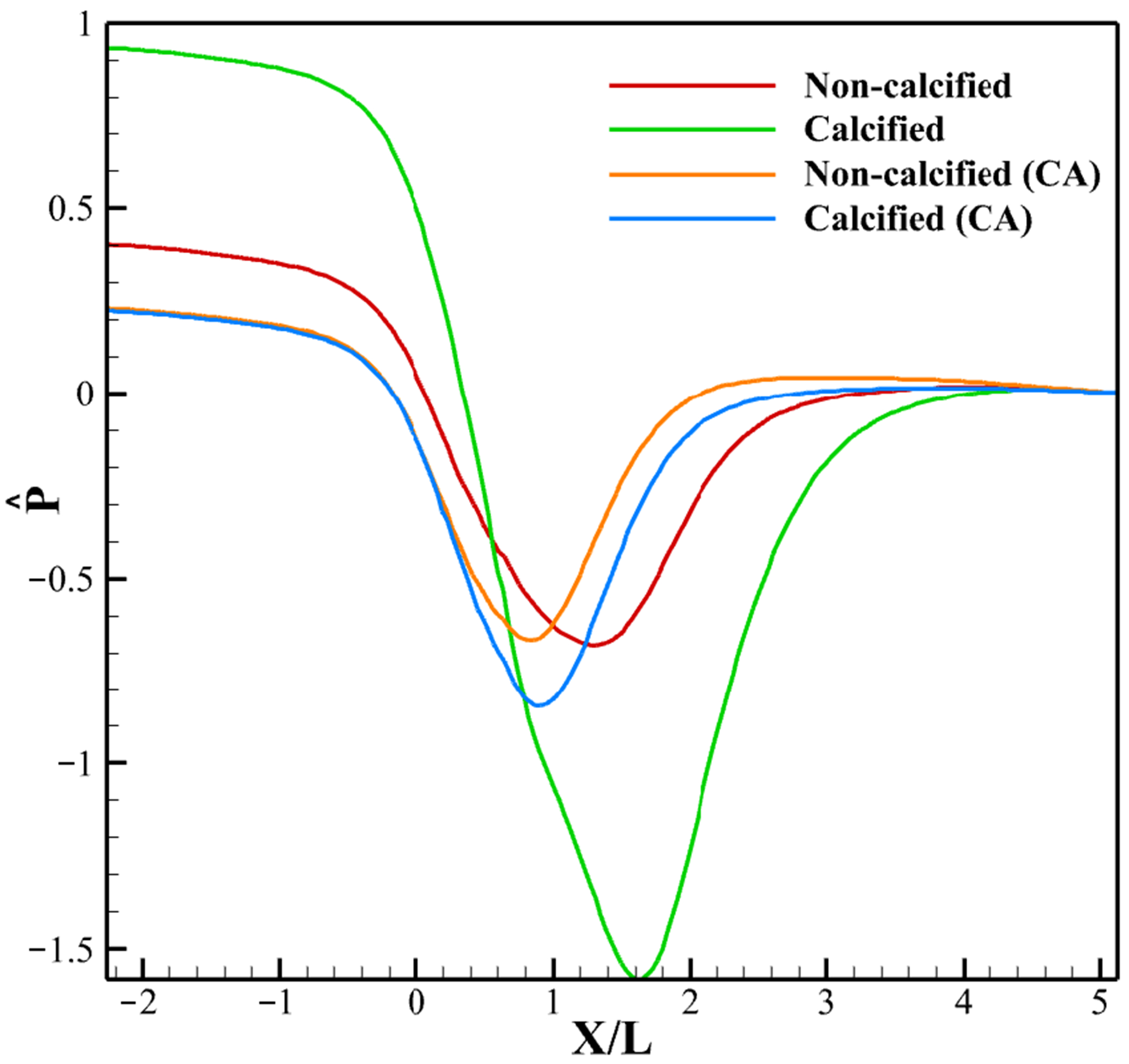
Pressure from inlet to outlet at peak systole.

Figure 13 shows the non-dimensionalized pressure $\left(\overset{ˆ}{P}\right)$ contours in the leaflet vicinity, with $\overset{ˆ}{P}$ defined as $\overset{ˆ}{P}=\frac{p-p_{\text{ref}}}{\rho U_{p}^{2}}$, where *p* is the pressure and $p_{\text{ref}}$ is the pressure at the exit. The corresponding centerline pressure distribution from inlet to outlet at peak systole is shown in Figure 14. In the no CA case, calcification transforms the gradual transvalvular pressure drop into an more abrupt decline, increasing the overall gradient and delaying pressure recovery downstream. With a CA, both non-calcified and calcified valves exhibit lower pressure drops than the no CA case. Calcification in this case causes a slight deepening of the downstream trough, with recovery occurring at nearly the same location as in the non-calcified CA case and earlier than in either no CA condition.

#### 3.2.3. Leaflet Profile and WSS

**Figure 15.**
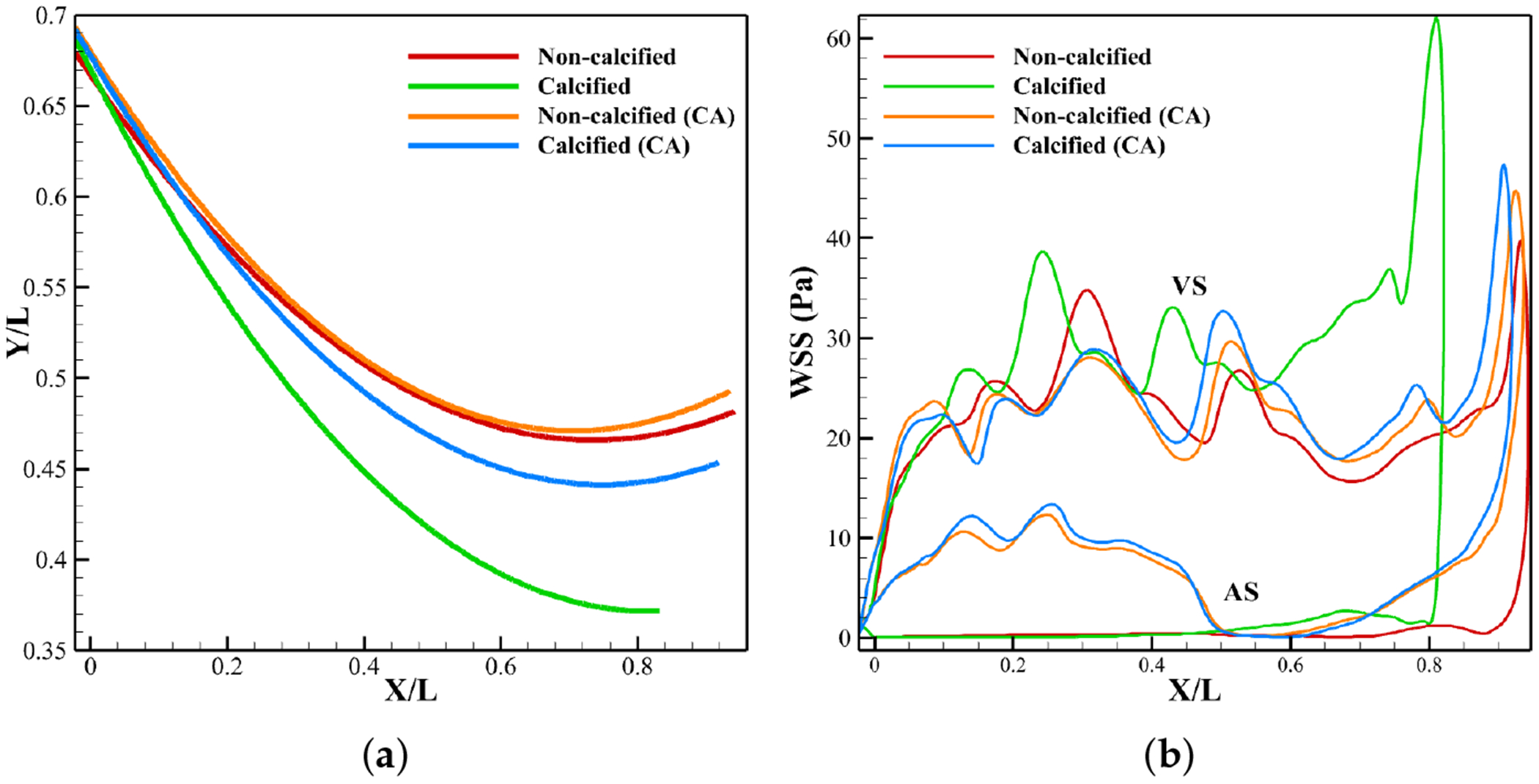
Leaflet profile and WSS distribution at peak systole. (**a**) Leaflet profile. (**b**) WSS.

Figure 15a presents the leaflet’s position at peak systole for the different cases.

Without CA, calcification shifts the leaflet downward, resulting in flatter and more restricted opening due to LDL accumulation (Figure 9a). With CA, the stiffened leaflet preserves its native curvature but opens slightly less than the non-calcified valve. Both non-calcified valves show similar curvature, with the CA case opening marginally wider, while the calcified CA valve maintains a greater opening than its calcified no CA counterpart.

Figure 15b shows the WSS at peak systole on the leaflet surface. Globally, without CA, WSS is higher on the ventricular side than the aortic side, consistent with Sadrabadi et al. [41]. On the aortic side, the non-calcified valve shows little variation, with a slight rise near the tip when calcified. On the ventricular side, WSS increases from the attachment to the tip, reaching higher levels when calcified due to accelerated flow near the orifice (Figure 6). With CA, the WSS patterns remain similar, though WSS on the aortic side is generally higher, while on the ventricular side it is lower from the attachment to midsection but rises near the tip due to close proximity to coronary suction.

### 3.3. Effect of LVOT Velocity Variation on LDL Distribution

Different segments of the aortic tract experience distinct LVOT velocity profiles due to the directionality of incoming flow [15]. To investigate this, four inlet profiles (anterior, medial, posterior, and lateral) were imposed (Figure 3), and their effects on LDL accumulation, downstream flow, and leaflet motion were analyzed.

#### 3.3.1. LDL Distribution, Velocity and Pressure

**Figure 16.**
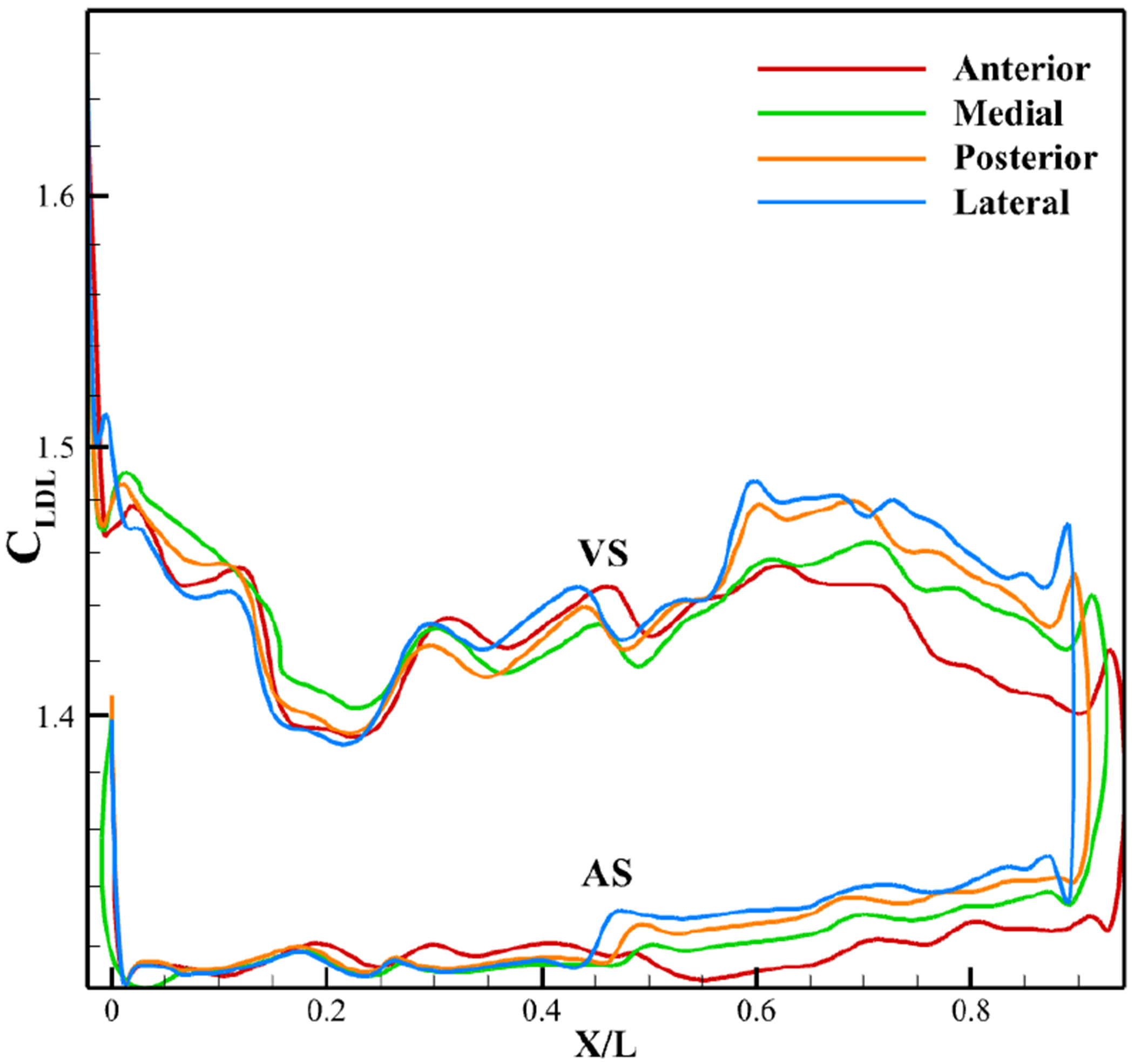
Average LDL variation across leaflet surface.

The impact of varying inlet velocity profiles (Figure 3) on leaflet LDL accumulation was analyzed and is presented in Figure 16. Across all cases, LDL accumulation at peak systole follows a similar pattern to the anterior profile from the attachment to the belly region on both the ventricular and aortic sides, but more pronounced differences emerge from the belly to the tip. In this region, LDL accumulation increases progressively with decreasing inlet velocity, reaching its maximum in the lateral section of the aortic valve.

**Figure 17.**
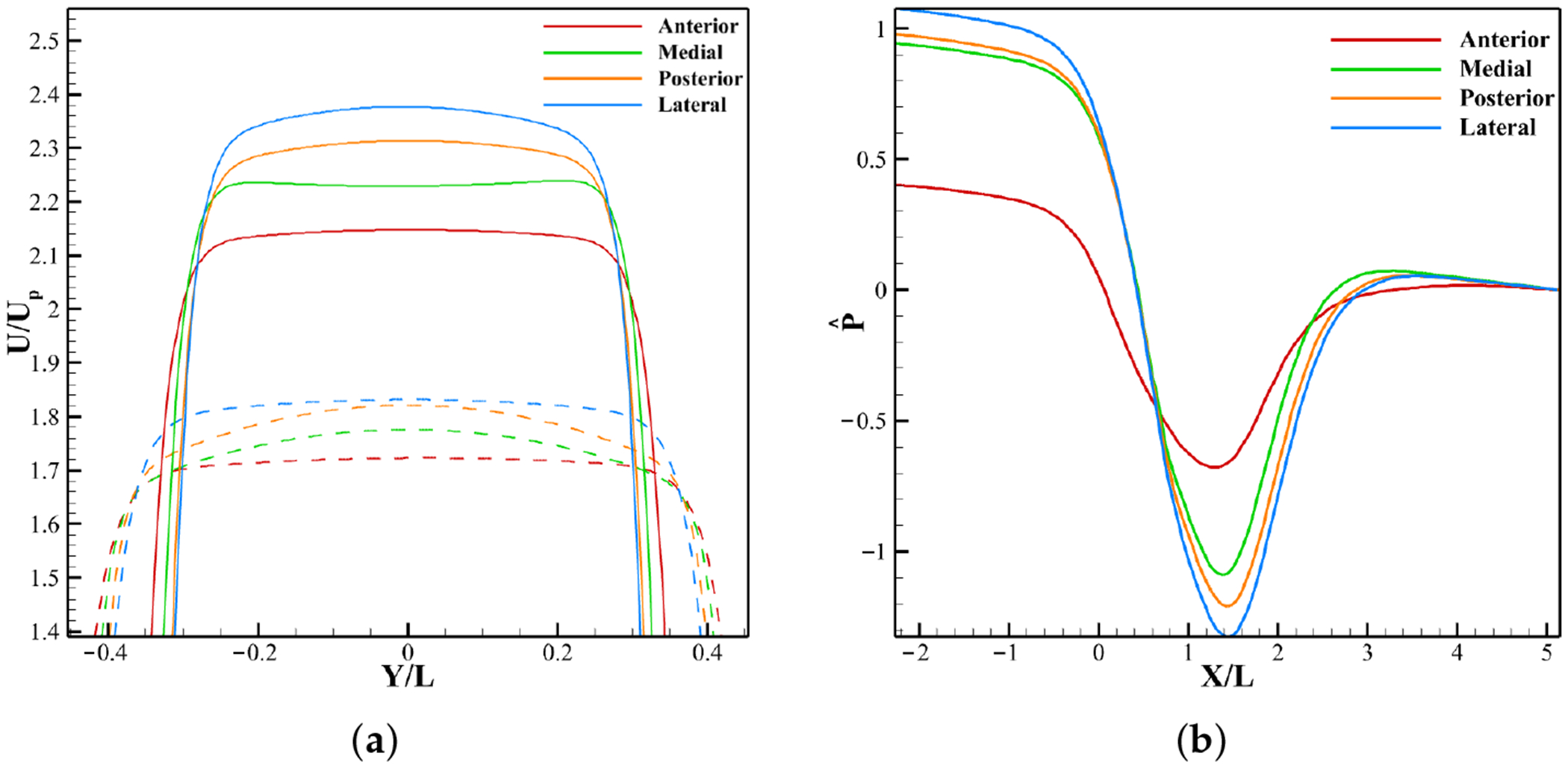
Effect of inlet velocity on velocity profile at aortic orifice and pressure at peak systole. (**a**) Jet velocity for non-calcified and calcified cases; (**b**) Pressure profile from inlet to outlet.

Figure 17a shows non-dimensionalized jet velocity profiles. Among non-calcified cases (dashed lines), the anterior profile has the highest inlet velocity but weakest jet, while medial, posterior, and lateral profiles show progressively rising jet peaks. With calcification (solid lines), all profiles exhibit stronger jets relative to their non-calcified states.

Figure 17b shows the non-dimensionalized pressure along the aortic tract for calcified cases. The anterior profile exhibits the smallest pressure drop due to its higher inlet velocity, whereas the medial and posterior profiles show greater drops and the lateral profile the largest. Pressure recovery in these cases occurs farther downstream than in the anterior profile.

#### 3.3.2. Leaflet Profile and WSS

**Figure 18.**
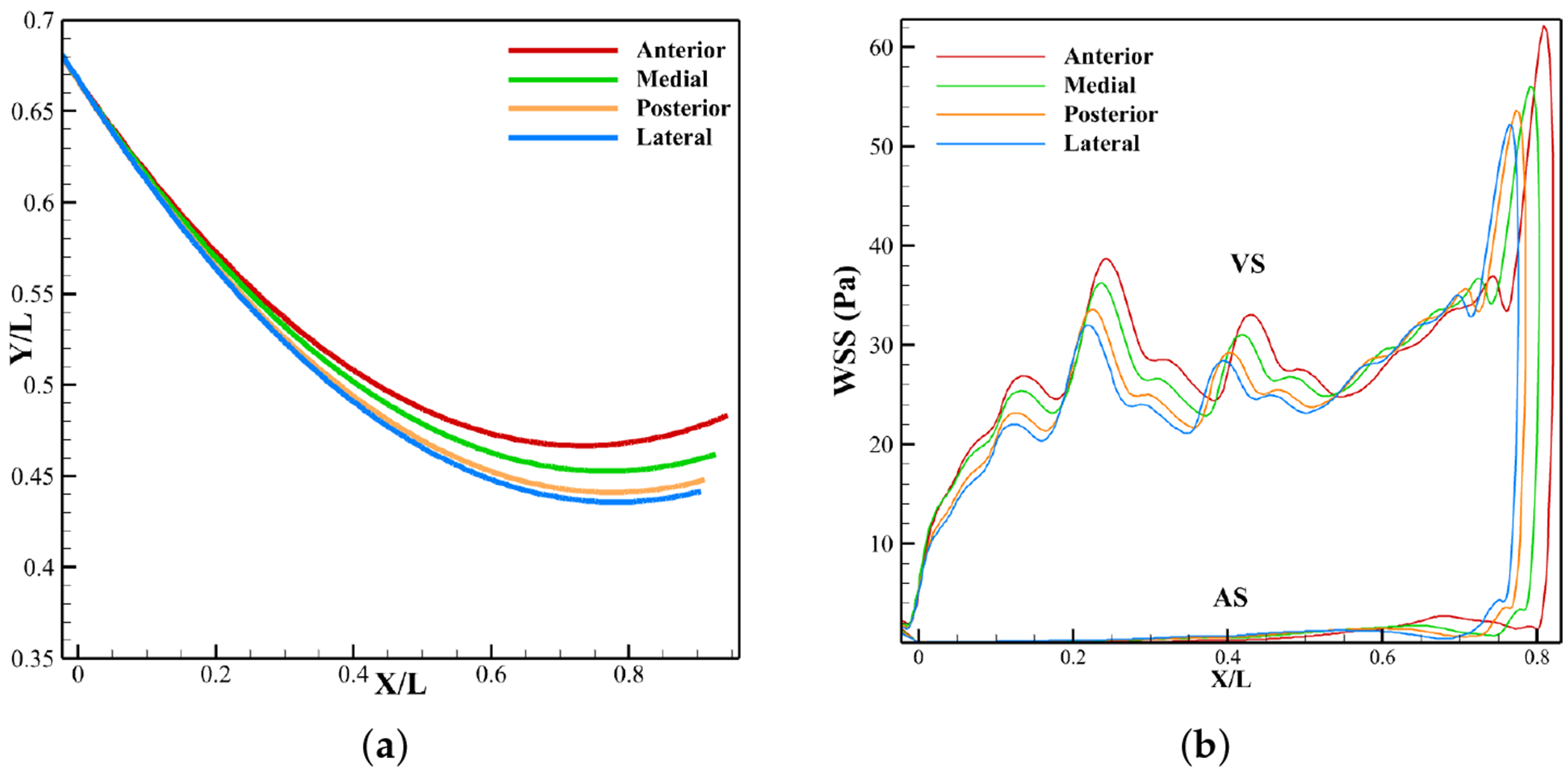
Effect of inlet velocity on leaflet profile and WSS on leaflet at peak systole. (**a**) Leaflet profile. (**b**) WSS.

Leaflet profiles at peak systole (Figure 18a) show similar overall curvature but differ near the tip, where LDL accumulation is most prominent. The anterior case achieves the widest opening, while the medial, posterior, and lateral cases exhibit progressively smaller half-openings.

Figure 18b shows WSS distributions along the leaflet surface at peak systole. All four profiles follow the same trend, with minimum WSS at the attachment and maximum at the ventricular tip. As inlet velocity decreases from anterior to lateral, overall WSS drops because reduced excursion exposes more surface area to the flow. Between the belly and tip, WSS remains nearly identical across cases, but elsewhere it rises progressively with lower inlet velocity. A slight increase in aortic side tip WSS also accompanies velocity reduction.

## 4. Discussion

This study examines how coronary artery flow and variations in LVOT velocity profiles influence leaflet-specific biomechanics, LDL accumulation and calcification.

The presence of coronary arteries plays an integral role in influencing aortic valve hemodynamics, with direct effects on leaflet excursion, LDL accumulation and retention, and the resulting patterns of calcification. Coronary artery presence lowers jet velocity (Figure 7a) and shortens the jet (Figures 6 and 7b), which directly reduces the transvalvular pressure drop (Figure 14) and allows earlier downstream flow recovery. It also markedly reduces vortex shedding at the leaflet edges (Figure 12), stabilizing the flow field and minimizing near-wall disturbances. These changes promote leaflet excursion (Figure 15a), a larger orifice area, and reduced energy loss during systole. This implies that the RCC and LCC undergo more pronounced excursion than the NCC, which is also consistent with clinical observations reported by Chen et al. [14].

Assessing LDL accumulation (Figures 8 and 9a) reveals that coronary flow reduces LDL accumulation substantially by enhancing circulation and washout (Figure 6). Globally, LDL deposition is greater on the ventricular side of the leaflet than on the aortic side for both cases with and without coronary artery, which is consistent with the observations reported by Sadrabadi et al. [41]. This asymmetry is primarily driven by the concentration polarization effect [50], wherein the high velocity systolic jet from the left ventricle directly impinges on the ventricular side, enhancing the convective transport of LDL particles toward the endothelial surface and exceeding the rate at which they can diffuse back into the bulk flow. Locally both cases show elevated LDL deposition near the attachment, on the ventricular side. This is linked to low velocity (Figure 6) and minimal WSS (Figure 15b), which limit clearance and promote buildup, while higher velocities and WSS toward the tip enhance convective transport and reduce accumulation [41]. On the aortic side, weaker flows and diffusion dominance yield steadier concentrations with mild polarization. These results suggest that calcification varies point-to-point across each leaflet and also differs among leaflets; specifically, the NCC shows greater LDL retention—and, thus, calcification—than the RCC or LCC, due to the absence of a nearby coronary ostium at the NCC, a pattern strongly supported by clinical studies [14,16].

Further analysis of the results suggest that WSS affects LDL retention. Although the ventricular side experiences higher near-wall LDL due to concentration polarization [50], the elevated WSS in this region promotes convective clearance and shorter residence times, thereby reducing the likelihood of oxidation and subsequent inflammatory responses, consistent with findings by Sadrabadi et al. [41], Soulis et al. [51], and Ha et al. [52]. In contrast, the aortic side generally shows lower tissue accumulation; however, the low flow environment and stagnation in its vicinity tend to promote LDL retention and oxidation. The resulting oxidized LDL has been associated with inflammatory signaling and osteogenic changes, thereby contributing to calcification within the valve tissue [19,20,48,49,53–56]. Coronary flow appears to mitigate these effects on both sides by promoting flow diversion, thereby enhancing LDL clearance and potentially lowering the risk of calcification.

The simulations with various velocity profiles, each representing distinct regions of the LVOT, show that higher LVOT velocities, as in the anterior region, enhance motion (Figure 18a), reduce the transvalvular pressure gradient (Figure 17b), and raise WSS (Figure 18b), thereby damping calcification effects (Figure 16). In contrast, lower velocity such as in the lateral region, raise jet velocity (Figure 17a), limit excursion, increase pressure drop, and reduce WSS.

When both LVOT velocity and coronary flow are taken into account, coronary washout has the dominant effect on LDL deposition (Figure 9a) but only a modest impact on excursion (Figure 15a). In contrast, LVOT velocity profiles drive larger differences in excursion (Figure 18a) and produce smaller variations in LDL accumulation (Figure 16). Therefore, the RCC, situated along the anterior region (Figure 4) and aided by both the highest local inlet velocities and coronary washout, experiences the greatest excursion and is least prone to calcification. The LCC, which lies predominantly in the lateral-posterior region, experiences the lowest inlet velocities and therefore the smallest excursion. However, coronary flow mitigates this disadvantage by enhancing LDL washout, so its calcification risk is higher than the RCC but lower than the NCC. The NCC, located in the medial-posterior region, experiences intermediate inlet velocities and excursion but has no coronary outflow. As a result, it lacks adequate LDL washout, accumulates the most lipid, and is therefore the most prone to calcification. These outcomes are in strong agreement with clinical studies by Veulemans et al. [57] and Gollmann et al. [16].

Although the simulations support clinical and computational findings [14,16,41,49–52,57,58] and illuminate the underlying physics, several limitations remain and should be addressed in future work. First, the analysis employed a simplified 2D model, which cannot fully capture the inherently 3D and complex behavior of the aortic valve. Nonetheless, this setup served as an effective first step to validate the framework, and future work will extend the model to 3D, patient-specific geometries to provide a more realistic representation of flow structures and leaflet dynamics. Second, coronary outflow was prescribed as a constant value rather than a fully time-dependent waveform. While this approach has been adopted in prior studies [36,37], future work will incorporate physiologically measured coronary waveforms. In addition, it will investigate how ostial height affects leaflet-specific calcification, potentially leading to different calcification levels between the RCC and LCC [14,58]. Another limitation of this study is the assumption of linear elastic leaflet properties. Future work will extend to hyperelastic valve models to better capture physiological realism. Finally, while the relationship between LDL concentration and leaflet stiffness was modeled using a simplified linear mapping, future refinements will explore nonlinear or experimentally derived constitutive laws to capture more physiologically accurate remodeling responses.

## 5. Conclusions

Considering the model employed in this study, both inlet flow dynamics and coronary outflow regulate the balance between biomechanical motion and lipid retention together. The RCC experiences the greatest excursion and least calcification, due to both strong inlet flow exposure and coronary washout. The LCC, with lower velocity and excursion, benefits from coronary flow, resulting in slightly higher calcification risk. The NCC, despite experiencing intermediate inlet velocities and greater excursion than the LCC, is most prone to calcification due to the absence of coronary outflow and limited LDL clearance.

## Data Availability

The data that support the findings of this study are available from the corresponding author upon reasonable request.

## Abbreviations

The following abbreviations are used in this manuscript:

CAVD: Calcific aortic valve disease
LCC: Left coronary cusp
RCC: Right coronary cusp
NCC: Non-coronary cusp
LVOT: Left ventricular outflow tract
LDL: Low-density lipoprotein
CA: Coronary artery

## References

[1] Hsieh G; Berman AN; Biery DW; Rizk T; Blankstein R The Current Landscape of Lipoprotein(a) in Calcific Aortic Valvular Disease. Curr. Opin. Cardiol 2021, 36, 542–548. 34397461 10.1097/HCO.0000000000000901 PMC8934151

[2] Bian W; Wang Z; Sun C; Zhang DM Pathogenesis and Molecular Immune Mechanism of Calcified Aortic Valve Disease. Front. Cardiovasc. Med 2021, 8, 765419. 35004882 10.3389/fcvm.2021.765419 PMC8734655

[3] Martin SS; Aday AW; Almarzooq ZI; Anderson CA; Arora P; Avery CL; Baker-Smith CM; Gibbs BB; Beaton AZ; Boehme AK; 2024 Heart Disease and Stroke Statistics: A Report of US and Global Data from the American Heart Association. Circulation 2024, 149, E347–E913. 38264914 10.1161/CIR.0000000000001209 PMC12146881

[4] Cai L; Hao Y; Ma P; Zhu G; Luo X; Gao H Fluid-structure interaction simulation of calcified aortic valve stenosis. Math. Biosci. Eng 2022, 19, 13172–13192. 36654041 10.3934/mbe.2022616

[5] Alharbi Y 2D Fluid-Structure Model of Aortic Valve Using a Derived Muscular Model Equation. In Proceedings of the 2023 International Conference on Bio Signals, Images, and Instrumentation (ICBSII), Chennai, India, 16–17 March 2023; pp. 1–4.

[6] Amindari A; Saltik L; Kirkkopru K; Yacoub M; Yalcin HC Assessment of calcified aortic valve leaflet deformations and blood flow dynamics using fluid-structure interaction modeling. Inform. Med. Unlocked 2017, 9, 191–199.

[7] Nowak M; Divo E; Adamczyk WP Fluid–Structure Interaction methods for the progressive anatomical and artificial aortic valve stenosis. Int. J. Mech. Sci 2022, 227, 107410.

[8] Kivi AR; Sedaghatizadeh N; Cazzolato BS; Zander AC; Nelson AJ; Roberts-Thomson R; Yoganathan A; Arjomandi M Hemodynamics of a stenosed aortic valve: Effects of the geometry of the sinuses and the positions of the coronary ostia. Int. J. Mech. Sci 2020, 188, 106015.

[9] Kivi AR; Sedaghatizadeh N; Cazzolato BS; Zander AC; Roberts-Thomson R; Nelson AJ; Arjomandi M Fluid structure interaction modelling of aortic valve stenosis: Effects of valve calcification on coronary artery flow and aortic root hemodynamics. Comput. Methods Programs Biomed 2020, 196, 105647. 32688138 10.1016/j.cmpb.2020.105647

[10] Oks D; Samaniego C; Houzeaux G; Butakoff C; Vázquez M Fluid–structure interaction analysis of eccentricity and leaflet rigidity on thrombosis biomarkers in bioprosthetic aortic valve replacements. Int. J. Numer. Methods Biomed. Eng 2022, 38, e3649. 10.1002/cnm.3649 36106918

[11] Raza-Taimuri M; Chen IY; Sadat H Hemodynamic Analysis of Non-Uniformly Calcified Aortic Valve Using a Partitioned Fluid-Structure Interaction Framework. Cardiovasc. Eng. Technol 2025, accepted . 10.1007/s13239-025-00811-z PMC12798824 41417418

[12] Amindari A; Kirkkopru K; Saltik L; Sunbuloglu E Effect of non-linear leaflet material properties on aortic valve dynamics—A coupled fluid-structure approach. Eng. Solid Mech 2021, 9, 123–136.

[13] Xie R; Han X; Xiong T; Chen M; Williams J; Lin P Numerical investigation of calcification effects on aortic valve motions and ambient flow characteristics. J. Fluids Struct 2024, 124, 104014.

[14] Chen IY; Vedula V; Malik SB; Liang T; Chang AY; Chung KS; Sayed N; Tsao PS; Giacomini JC; Marsden AL; Preoperative Computed Tomography Angiography Reveals Leaflet-Specific Calcification and Excursion Patterns in Aortic Stenosis. Circ. Cardiovasc. Imaging 2021, 14, 1122–1132. 34915729 10.1161/CIRCIMAGING.121.012884 PMC9206593

[15] Kupari M; Hekali P; Poutanen VP Cross sectional profiles of systolic flow velocities in left ventricular outflow tract of normal subjects. Heart 1995, 74, 176–181. 10.1136/hrt.74.1.34 PMC483943 7662450

[16] Gollmann-Tepeköylü C; Nägele F; Engler C; Stoessel L; Zellmer B; Graber M; Hirsch J; Pölzl L; Ruttmann E; Tancevski I; Different calcification patterns of tricuspid and bicuspid aortic valves and their clinical impact. Interact. Cardiovasc. Thorac. Surg 2022, 35, ivac274. 36383200 10.1093/icvts/ivac274 PMC10906007

[17] Wanchaitanawong W; Kanjanavanit R; Srisuwan T; Chootipongchaivat S; Laksanabunsong P Diagnostic role of aortic valve calcium scoring in various etiologies of aortic stenosis. Sci. Rep 2023, 13, 8019. 37198243 10.1038/s41598-023-34118-7 PMC10192370

[18] Zhang B; Salaun E; Côté N; Wu Y; Mahjoub H; Mathieu P; Dahou A; Zenses AS; Clisson M; Pibarot P; Association of Bioprosthetic Aortic Valve Leaflet Calcification on Hemodynamic and Clinical Outcomes. J. Am. Coll. Cardiol 2020, 76, 1737–1748. 33032735 10.1016/j.jacc.2020.08.034

[19] Demer LL; Tintut Y Vascular calcification: Pathobiology of a multifaceted disease. Circulation 2008, 117, 2938–2948. 18519861 10.1161/CIRCULATIONAHA.107.743161 PMC4431628

[20] Tsimikas S; Karwatowska-Prokopczuk E; Gouni-Berthold I; Tardif JC; Baum SJ; Steinhagen-Thiessen E; Shapiro MD; Stroes ES; Moriarty PM; Nordestgaard BG; Lipoprotein(a) Reduction in Persons with Cardiovascular Disease. N. Engl. J. Med 2020, 382, 244–255. 31893580 10.1056/NEJMoa1905239

[21] Lerman DA; Prasad S; Alotti N Calcific Aortic Valve Disease: Molecular Mechanisms and Therapeutic Approaches. Eur. Cardiol. Rev 2015, 10, 108–112. 10.15420/ecr.2015.10.2.108 PMC4888946 27274771

[22] The OpenFOAM Foundation. OpenFOAM: The Open Source CFD Toolbox, Version 11; The OpenFOAM Foundation: London, UK, 2024.

[23] Dhondt G CalculiX: A Free Software Three-Dimensional Structural Finite Element Program, Version 2.21; MTU Aero Engines: Munich, Germany, 2024.

[24] Bungartz HJ; Lindner F; Gatzhammer B; Mehl M; Scheufele K; Shukaev A; Uekermann B preCICE—A Fully Parallel Library for Multi-Physics Surface Coupling. Comput. Fluids 2016, 141, 250–258.

[25] Huang Z; Merkle C; Abdallah S; Tarbell J Numerical simulation of unsteady laminar flow through a tilting disk heart valve: Prediction of vortex shedding. J. Biomech 1994, 27, 391–402. 8188720 10.1016/0021-9290(94)90015-9

[26] Arminio M; Carbonaro D; Morbiducci U; Gallo D; Chiastra C Fluid-structure interaction simulation of mechanical aortic valves: A narrative review exploring its role in total product life cycle. Front. Med. Technol 2024, 6, 1399729. 39011523 10.3389/fmedt.2024.1399729 PMC11247014

[27] Bos F; Matijašević D; Terze Z; Oudheusden B; Bijl H OpenFOAM mesh motion using Radial Basis Function interpolation In Proceedings of the 5th European Congress on Computational Methods in Applied Sciences and Engineering (ECCOMAS 2008), Venice, Italy, 30 June–5 July 2008.

[28] Sun W; Martin C; Pham T Computational modeling of cardiac valve function and intervention. Annu. Rev. Biomed. Eng 2014, 16, 53–76. 24819475 10.1146/annurev-bioeng-071813-104517 PMC5481457

[29] Cataloglu A; Gould P; Clark R Validation of a simplified mathematical model for the stress analysis of human aortic heart valves. J. Biomech 1975, 8, 347–348. 1184606 10.1016/0021-9290(75)90088-3

[30] Richter T Fluid-Structure Interactions: Models, Analysis and Finite Elements; Lecture Notes in Computational Science and Engineering; Springer: Berlin/Heidelberg, Germany, 2017; Volume 118.

[31] Badia S; Quaini A; Quarteroni A Splitting Methods Based on Algebraic Factorization for Fluid-Structure Interaction. SIAM J. Sci. Comput 2008, 30, 1778–1805.

[32] Bazilevs Y; Calo VM; Hughes TJ; Zhang Y Isogeometric Fluid-Structure Interaction: Theory, Algorithms, and Computations. Comput. Mech 2008, 43, 3–37.

[33] Geuzaine C; Remacle JF Gmsh: A 3-D finite element mesh generator with built-in pre- and post-processing facilities. Int. J. Numer. Methods Eng 2009, 79, 1309–1331.

[34] Halevi R; Hamdan A; Marom G; Mega M; Raanani E; Haj-Ali R Progressive aortic valve calcification: Three-dimensional visualization and biomechanical analysis. J. Biomech 2015, 48, 489–497. 25553668 10.1016/j.jbiomech.2014.12.004

[35] Tian FB; Dai H; Luo H; Doyle FJ; Rousseau B Fluid-structure interaction involving large deformations: 3D simulations and applications to biological systems. J. Comput. Phys 2014, 258, 451–469. 10.1016/j.jcp.2013.10.047 PMC3884079 24415796

[36] Al-Jughiman MK; Al-Omair MA Modelling coronary flow after the Norwood operation: Influence of a suggested novel technique for coronary transfer. Glob. Cardiol. Sci. Pract 2018, 2018, 7. 29644234 10.21542/gcsp.2018.7 PMC5857063

[37] González-Suárez A; Pérez JJ; O’Brien B; Elahi A In Silico Modelling to Assess the Electrical and Thermal Disturbance Provoked by a Metal Intracoronary Stent during Epicardial Pulsed Electric Field Ablation. J. Cardiovasc. Dev. Dis 2022, 9, 458. 36547455 10.3390/jcdd9120458 PMC9784210

[38] Flemister DC; Hatoum H; Guhan V; Zebhi B; Lincoln J; Crestanello J; Dasi LP Effect of Left and Right Coronary Flow Waveforms on Aortic Sinus Hemodynamics and Leaflet Shear Stress: Correlation with Calcification Locations. Ann. Biomed. Eng 2020, 48, 2796–2808. 33145675 10.1007/s10439-020-02677-9 PMC11022940

[39] Kolandavel MK; Fruend ET; Ringgaard S; Walker PG The effects of time varying curvature on species transport in coronary arteries. Ann. Biomed. Eng 2006, 34, 1820–1832. 17051428 10.1007/s10439-006-9188-3 PMC1705526

[40] Obaid D; Molina J; Ademiloye A A New Open-source Solver for Early Detection of Atherosclerosis Based On Hemodynamics and LDL Transport Simulation. Eng. Rep 2024, 6, e12955.

[41] Sadrabadi MS; Hedayat M; Borazjani I; Arzani A Fluid-structure coupled biotransport processes in aortic valve disease. J. Biomech 2021, 117, 110239. 33515904 10.1016/j.jbiomech.2021.110239

[42] Butcher JT; Nerem RM Valvular endothelial cells and the mechanoregulation of valvular pathology. Philos. Trans. R. Soc. B Biol. Sci 2007, 362, 1445–1457. 10.1098/rstb.2007.2127 PMC2440407 17569641

[43] Yang N; Vafai K Modeling of low-density lipoprotein (LDL) transport in the artery—Effects of hypertension. Int. J. Heat Mass Transf 2006, 49, 850–867.

[44] Soulis JV; Giannoglou GD; Dimitrakopoulou M; Papaioannou V; Logothetides S; Mikhailidis DP Low-density lipoprotein concentration in the normal left coronary artery tree. Clin. Hemorheol. Microcirc 2008, 39, 251–261.

[45] Yazdani SK; Ku DN Oscillating LDL accumulation in normal human aortic arch. Biorheology 2010, 47, 303–319.

[46] Nematollahi A; Shirani E; Mirzaee I; Sadeghi M Numerical simulation of LDL particles mass transport in human carotid artery under steady state conditions. Sci. Iran 2012, 19, 519–524.

[47] Pletcher MJ; Bibbins-Domingo K; Liu K; Sidney S; Lin F; Vittinghoff E; Hulley SB Nonoptimal lipids commonly present in young adults and coronary calcium later in life: The CARDIA (Coronary Artery Risk Development in Young Adults) study. Ann. Intern. Med 2010, 153, 137–146. 20679558 10.1059/0003-4819-153-3-201008030-00004 PMC3468943

[48] Weinberg EJ; Mack PJ; Schoen FJ; García-Cardeña G; Mofrad MRK Hemodynamic Environments from Opposing Sides of Human Aortic Valve Leaflets Evoke Distinct Endothelial Phenotypes In Vitro. Cardiovasc. Eng 2010, 10, 5–11. 20107896 10.1007/s10558-009-9089-9 PMC2837826

[49] Yip CYY; Simmons CA The aortic valve microenvironment and its role in calcific aortic valve disease. Cardiovasc. Pathol 2011, 20, 177–182. 21256052 10.1016/j.carpath.2010.12.001

[50] Vincent PE; Weinberg PD Flow-dependent concentration polarization and the endothelial glycocalyx layer: Multi-scale aspects of arterial mass transport and their implications for atherosclerosis. Biomech. Model. Mechanobiol 2014, 13, 313–326. 23836008 10.1007/s10237-013-0512-1

[51] Soulis J; Giannoglou G; Dimitrakopoulou M; Papaioannou V; Logothetides S; Mikhailidis D Influence of oscillating flow on LDL transport and wall shear stress in the normal aortic arch. Open Cardiovasc. Med. J 2009, 3, 128–142. 19834577 10.2174/1874192400903010128 PMC2761669

[52] Ha H; Kim GB; Kweon J; Lee SJ; Kim Y; Kim N; Yang DH The influence of the aortic valve angle on the hemodynamic features of the thoracic aorta. Sci. Rep 2016, 6, 32316. 27561388 10.1038/srep32316 PMC4999809

[53] Cao K; Sucosky P Aortic valve leaflet wall shear stress characterization revisited: Impact of coronary flow. Comput. Methods Biomech. Biomed. Eng 2016, 20, 468–470. 10.1080/10255842.2016.1244266 27712083

[54] Hayashi H; Akiyama K; Itatani K; DeRoo S; Sanchez J; Ferrari G; Colombo PC; Takeda K; Wu IY; Kainuma A; A novel in vivo assessment of fluid dynamics on aortic valve leaflet and its relationship with leaflet thickening. Sci. Rep 2020, 10, 12345. 32003907 10.1111/echo.14596 PMC8151665

[55] Yap CH; Saikrishnan N; Tamilselvan G; Ajit P Experimental measurement of dynamic fluid shear stress on the aortic surface of the aortic valve leaflet. Ann. Biomed. Eng 2011, 39, 799–808.

[56] Rosakis G; Gharib M The Influence of Valve Leaflet Stiffness Variability on Aortic Wall Shear Stress and LDL Deposition. J. Biomech 2022, 120, 110357. 10.1007/s10439-021-02899-5 34993697

[57] Veulemans V; Piayda K; Maier O; Bosbach G; Polzin A; Hellhammer K; Afzal S; Klein K; Dannenberg L; Zako S; Aortic valve calcification is subject to aortic stenosis severity and the underlying flow pattern. Heart Vessel. 2021, 36, 242–251. 10.1007/s00380-020-01688-9 PMC7843559 32894344

[58] Cheng CL; Chang HH; Huang PJ; Wang WC; Lin SY Different Calcification Stage in Each Cusp of a Calcified Tricuspid Aortic Valve. Circ. J 2017, 81, 1953–1955. 28442637 10.1253/circj.CJ-17-0129

